# Solid-State Chemical Recycling of Polycarbonates to Epoxides and Carbon Dioxide Using a Heterodinuclear Mg(II)Co(II) Catalyst

**DOI:** 10.1021/jacs.2c06937

**Published:** 2022-09-28

**Authors:** Thomas M. McGuire, Arron C. Deacy, Antoine Buchard, Charlotte K. Williams

**Affiliations:** † Department of Chemistry, Chemistry Research Laboratory, University of Oxford, 12 Mansfield Rd, Oxford, OX1 3TA, U.K.; ‡ Department of Chemistry, University of Bath, Centre for Sustainable and Circular Technologies, Claverton Down, Bath BA2 7AY, U.K.

## Abstract

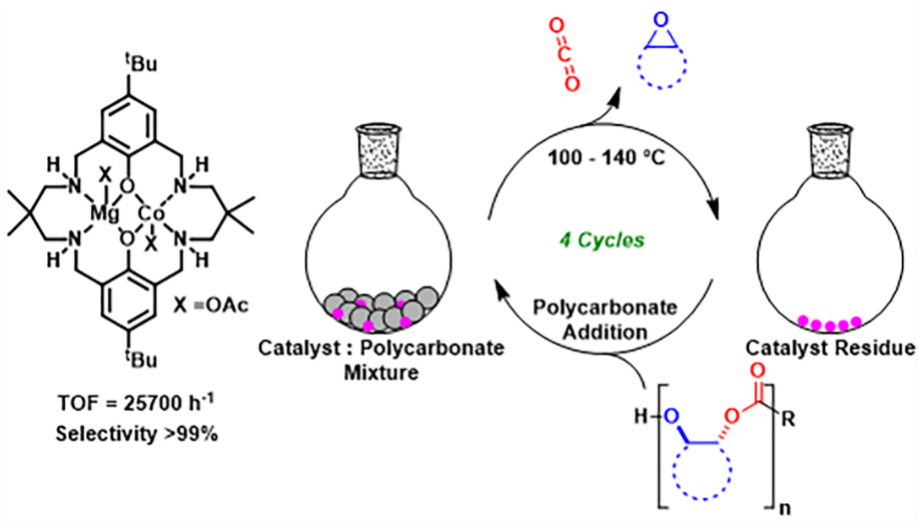

Polymer chemical recycling to monomers (CRM) could help improve polymer sustainability, but its implementation requires much better understanding of depolymerization catalysis, ensuring high rates and selectivity. Here, a heterodinuclear [Mg(II)Co(II)] catalyst is applied for CRM of aliphatic polycarbonates, including poly(cyclohexene carbonate) (PCHC), to epoxides and carbon dioxide using solid-state conditions, in contrast with many other CRM strategies that rely on high dilution. The depolymerizations are performed in the solid state giving very high activity and selectivity (PCHC, TOF = 25700 h^–1^, CHO selectivity >99 %, 0.02 mol %, 140 °C). Reactions may also be performed in air without impacting on the rate or selectivity of epoxide formation. The depolymerization can be performed on a 2 g scale to isolate the epoxides in up to 95 % yield with >99 % selectivity. In addition, the catalyst can be re-used four times without compromising its productivity or selectivity.

## Introduction

Polymer chemical recycling to monomer (CRM) could improve sustainability by reducing wastes and limiting embedded emissions in virgin monomer production. The method forms pure monomers and allows for repolymerization to polymers that are equivalent to the original materials, thereby overcoming the deteriorating property profiles associated with other recycling technologies.^1,2^ All polymers have a ceiling temperature above which depolymerization to monomer is thermodynamically feasible, but there are depolymerization activation energies to overcome, and, in practice, CRM remains a major challenge. CRM catalysis needs better understanding of how to reduce depolymerization barriers, deliver high monomer selectivity, and minimize energy inputs. Depolymerizations of aliphatic polyesters/carbonates to cyclic esters/carbonates are front-runner recycling to monomer reactions.^3−8^ High monomer conversions and selectivity are achieved by careful manipulation of the process conditions, in particular, exploiting high dilution, and using special monomer structures, most commonly by applying low ring-strain heterocycles.^9−14^ The approach is to bias depolymerization equilibria: the trade-off is less favorable forward polymerization thermodynamics. Such polymers may also have low overall thermal stability, which may complicate their processing and use. An alternative strategy lies in the aliphatic polycarbonate depolymerization to epoxides and carbon dioxide, which is expected to be strongly entropically favored and driven by gaseous carbon dioxide removal.^15,16^ A critical factor in these depolymerizations is to limit 5-membered cyclic carbonate formation since those compounds are thermodynamically more stable than the polymers and cannot be easily repolymerized.

**Figure 1:**
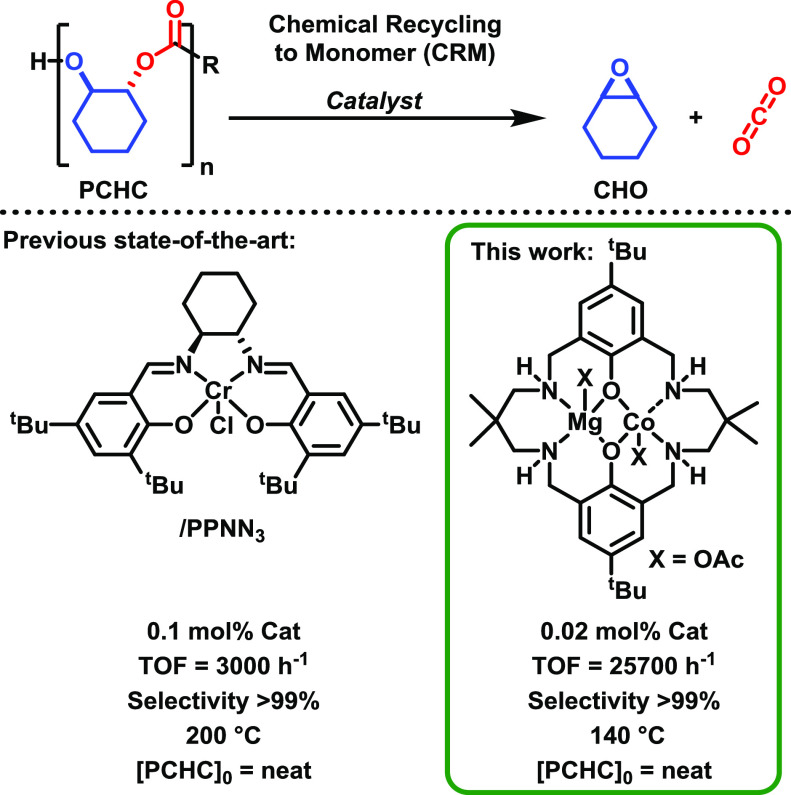
Catalysts for depolymerization of poly(cyclohexene carbonate) (PCHC) to cyclohexene oxide (CHO) and CO_2_.

In 2013, Darensbourg and co-workers pioneered poly(cyclopentene carbonate) (PCPC) depolymerization to cyclopentene oxide, using a Cr(III)-salen/^*n*^Bu_4_NN_3_ catalyst system, achieving 92 % selectivity.^17,18^ Subsequently, others reported a few more polycarbonate depolymerizations using poly(*N*-heterocyclic epoxide carbonates)^19,20^ or poly(limonene carbonate) (PLC).^21,22^ The CRM of poly(cyclohexene carbonate) (PCHC), the most widely studied carbon dioxide-derived polycarbonate, remained, until very recently, a challenge (Figure 1).^23−25^

Cyclohexene oxide/CO_2_ ROCOP is the benchmark for catalyst comparisons and PCHC shows a high glass transition temperature (∼120 °C) and tensile modulus (3600 MPa).^28^ It has a broad processing temperature range (>100 °C), and thermal degradation begins at temperatures of >240 °C.^29−31^ PCHC-containing block polymers show promise as toughened plastics, elastomers, and adhesives.^32^ Thus, achieving its chemical recycling to monomer is important; earlier this year, we reported a Mg(II)Mg(II) catalyst, applied in *para*-xylene solution, which delivered very high CHO selectivity (98 %) and a turn-over-frequency (TOF) of 150 h^–1^ (0.33 mol % catalyst, 120 °C, [PCHC]_0_ = 1 M) (Figure 1).^26^ As this manuscript was submitted, Lu and co-workers reported PCHC pyrolysis using a Cr(III)(salen)Cl/PPNN_3_ catalyst system, which showed really excellent rates, albeit at high temperature (Figure 1, 0.2 mol % catalyst, TOF = 3000 h^–1^, 200 °C).^27^ A decomposed di-Zn(II) catalyst was also reported for PCHC depolymerization.^33^ Our focus is on improving catalytic activity, selectivity, and applicability in PCHC depolymerization. Solid-state depolymerizations were targeted since obviating organic solvents could be beneficial both for environmental impact and process scale-up. To increase rates, the principle of polymerization microscopic reversibility directs toward testing of a faster forward polymerization catalyst. In 2020, our team reported a synergic heterodinuclear Mg(II)Co(II) catalyst for CHO/CO_2_ ROCOP, showing significantly greater activity than the Mg(II)Mg(II) species, and thus this heterodinuclear catalyst was a candidate for investigation.^34^

## Results and Discussion

**Figure 2:**
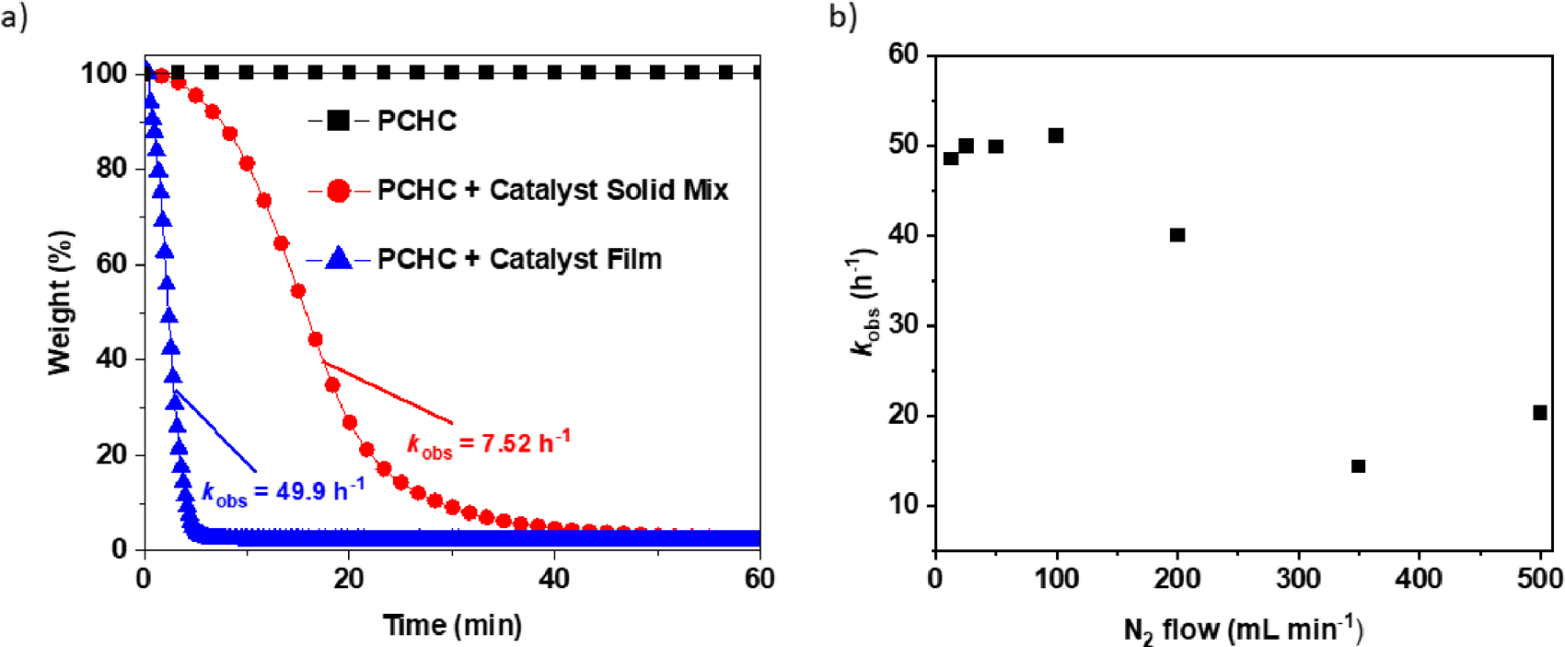
(a) Solid-state PCHC depolymerizations using Mg(II)Co(II) (1:300), at 140 °C, showing mass loss over time. Black = PCHC, red = Mg(II)Co(II):PCHC, 1:300, mixed with a mortar and pestle; blue = Mg(II)Co(II):PCHC, 1:300, film (solvent cast), CHO selectivity > 99%. Depolymerization rate coefficients, *k*_obs_, are obtained by exponential fits to the data. (b) Plot showing depolymerization rate coefficients, *k*_obs_, vs nitrogen flow rate (mL min^–1^) at 140 °C for polymer films (i.e., Mg(II)Co(II):PCHC, 1:300, film).

Dihydroxy-telechelic PCHC was prepared by ROCOP of CHO and CO_2_ (*M*_n,SEC_ = 8000 g mol^–1^, *Đ*_M_ = 1.07, *M*_n,NMR_ = 5400 g mol^–1^). Solid-state PCHC depolymerizations were achieved by mixing the polymer and catalyst with the reaction being monitored using thermogravimetric analysis (TGA). Samples were heated, under nitrogen or air flow, with either isothermal or variable temperature conditions, and PCHC mass loss was monitored over time (see SI for experimental details). First, solid samples of Mg(II)Co(II):PCHC, 1:300, were mixed using a mortar and pestle. These specimens were successfully depolymerized at 140 °C, under a nitrogen flow, resulting in an 80% PCHC mass loss in only 20 min (Table 1, entry 1). The catalyst achieved a turn-over-frequency (TOF) of 900 h^–1^. The mass loss data fit an exponential decay and have a pseudo first order rate coefficient for depolymerization, *k*_obs_ = 7.52 h^–1^, indicative of a first order dependence on polymer mass (Figure 2a). Repeat experiments showed excellent reproducibility with near perfect overlay of data (Figure 2, Figure S2). Depolymerization rates were equivalently high using either nitrogen or air flow, the latter tolerance being notable as many polymerization catalysts are air-sensitive. (Figure S3). It is emphasized that these reactions are depolymerizations not merely polymer decompositions: the PCHC sample used has a significantly higher on-set for thermal decomposition (*T*_d,5%_) of 255 °C. Also, the catalyst is thermally stable, with a *T*_d10%_ of 365 °C. Control samples of PCHC, without any catalyst, showed no degradation over 24 h under equivalent conditions using N_2_ or air (Figure 2a). To analyze the depolymerization product(s) formed under both N_2_ and air, a cold-trap condensed the liquid product, which by NMR spectroscopy was pure cyclohexene oxide in both cases (90% yield, Figures S4–S6). The residual mass, after depolymerization, of <3 wt % corresponds to the catalyst loading. CO_2_ was also detected through TGA-MS experiments, indicating that both epoxide and CO_2_ are extruded in the depolymerization (Figure S7).

**Table 1:** Data for PCHC Depolymerizations using a Mg(II)Co(II) Catalyst in the Solid State with some Solution-State Literature Benchmark Catalysts^a^

entry	catalyst	temperature (°C)	[PCHC]_0_:[cat]_0_	time (s)^b^	TOF (h^–1^)^c^	mass loss rate (kg g^–1^ h^–1^)^d^	*k*_obs_ (h^–1^)^e^
1^f^	Mg(II)Co(II)	140	300:1	1327	900(±50)	0.17 (±0.09)	7.52(±0.01)
2	Mg(II)Co(II)	140	300:1	109	6000(±300)	1.12(±0.06)	49.9(±0.1)
3	Mg(II)Mg(II)	140	300:1	557	1200(±60)	0.22(±0.01)	9.72(±0.01)
4	Mg(II)Co(II)	140	1000:1	155	13900(±700)	2.64(±0.1)	38.7(±0.1)
5	Mg(II)Co(II)	140	2500:1	241	22400(±1100)	4.23(±0.2)	22.6(±0.02)
6	Mg(II)Co(II)	140	5000:1	420	25700(±1300)	4.86(±0.2)	13.8(±0.02)
7	Mg(II)Co(II)	140	10,000:1	1735	12400(±600)	2.35(±0.1)	2.62(±0.02)
8	Mg(II)Co(II)	100	300:1	2653	200(±10)	0.05(±0.003)	2.29(±0.002)
9	Mg(II)Co(II)	110	300:1	1060	600(±30)	0.11(±0.006)	4.76(±0.001)
10	Mg(II)Co(II)	120	300:1	370	1800(±90)	0.33(±0.02)	14.7(±0.02)
11	Mg(II)Co(II)	130	300:1	201	3200(±160)	0.61(±0.03)	24.8(±0.1)
12^26^	Mg(II)Mg(II)	120	300:1	1200	150	0.020^g^	0.407
13^27^	Cr(III)/PPNN_3_	200	1000:1	1200	3000	0.35^h^	-
14^27^	Cr(III)/PPNN_3_	140	500:1	36,000	2.5	0.00059^h^	-

a See SI for details of experimental setup, all TGA experiments run to >99% mass loss, CHO selectivity > 99%.

b Interval of time from 20 to 80% mass loss of the polymer.

c TOF = moles of PCHC consumed (20–80% conversion)/moles of catalyst/time (see SI). Average error taken from repeat runs (<5%).

d Mass loss rate = mass PCHC consumed (20–80% conversion)/catalyst mass/time.

e *k*_obs_ = gradient of linear fitting of the logarithm of %polymer mass vs time (Figures S8–18).

f Polymer:catalyst mixed by pestle and mortar.

g Values calculated from ref (26).

h Values calculated from ref (27).

Sample mixing was investigated with catalyst/polymer mixtures dissolved in minimum solvent (THF or toluene) and the solvent removed *in-vacuo* to form a polymer film (see SI). These samples, which have the same catalyst loading (Mg(II)Co(II):PCHC, 1:300), showed much faster rates, achieving 80% PCHC depolymerization in just 3.5 mins corresponding to a TOF of 6000 h^–1^. The data also fit exponential depolymerization decays, and the rate coefficient, *k*_obs_ = 49.9 h^–1^, is ∼7 times greater than samples mixed with a mortar and pestle (Table 1, Entry 2, Figure 2). Furthermore, the film depolymerizations occurred with improved initiation times as compared with both the blended solid-state and solution-phase reactions (<1 min vs >10 mins).^26^ It is proposed that the faster rates arise from better catalyst dispersion in the polymer and the high film surface area. The nitrogen flow rate also influenced the catalysis with the best rates arising from moderate flows (Figure 2b and Table S2). At nitrogen flows from 25 to 100 mL min^–1^, the activity was consistently high and data showed exponential decays, with rate constants (*k*_obs_) of ∼50 ± 1 h^–1^. At higher flow rates, 100–500 mL min^–1^, rates slowed perhaps due to CO_2_ and/or CHO release inhibition. This is attributed to the high flow rates causing overpressure of the reaction furnace and preventing release of the co-monomers from the TGA crucible.

The Mg(II)Co(II) shows 5× greater activity than the Mg(II)Mg(II) catalyst, when tested under the solid-state conditions. The Mg(II)Mg(II) catalyst achieved a TOF = 1200 h^–1^, while the Mg(II)Co(II) catalyst had a TOF of 6000 h^–1^ (Table 1, entry 3). This finding suggests that faster polymerization catalysts are faster depolymerization catalysts. It also indicates that depolymerization catalysis can be accelerated by exploiting heterodinuclear synergy.^34,35^ To understand the limits of catalyst tolerance, the loading of Mg(II)Co(II) was decreased from 1:300 to 1:10000. At a 1:5000 catalyst:PCHC loading, complete depolymerization was achieved corresponding to a TOF of 25700 h^–1^ (*k*_obs_ = 13.8 h^–1^, Figure S20). Such an activity is remarkable since it would correspond to a mass loss rate of 5 kg PCHC per gram catalyst per hour. At the lowest loadings, depolymerization still occurred although with slightly lower activity, (1:10000, TOF = 12400 h^–1^), which is attributed to some catalyst decomposition. Compared with the next best other depolymerization catalyst (Cr(salen)/PPNCl) under as close to equivalent conditions as possible,^27^ the Mg(II)Co(II) catalyst shows >5000× greater activity at one tenth the catalyst loading (Table 1, entry 14).

**Figure 3:**
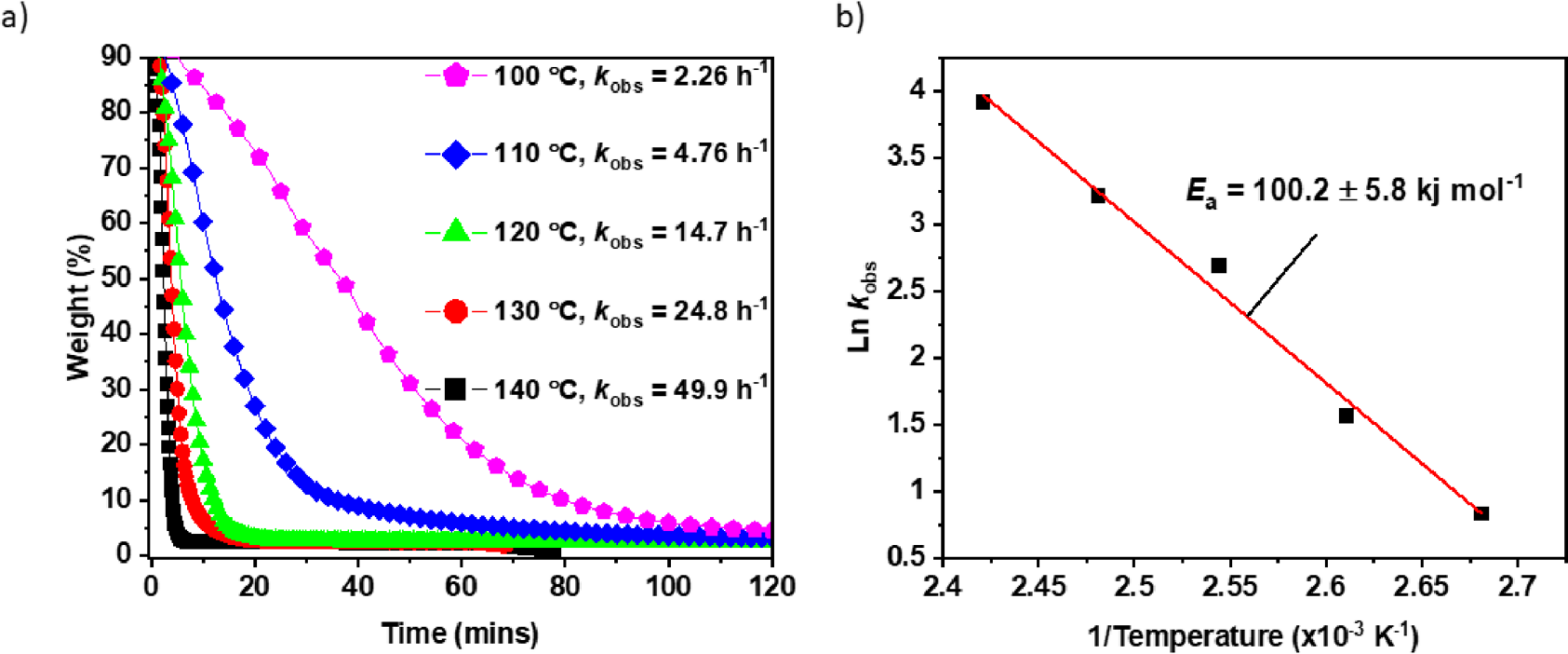
(a) Solid state PCHC depolymerizations using Mg(II)Co(II) catalyst (1:300) at temperatures from 100 to 140 °C. (b) Arrhenius plot for PCHC depolymerizations (ln(*k*_obs_) vs 1/*T*) using data collected from 100 to 140 °C.

Successful depolymerization catalysis was achieved at temperatures above 100 °C, yielding CHO as the sole product in all cases (Figure 3a). As expected, the catalytic activity increased with temperature from TOF = 200 h^–1^ (*k*_obs_ 0.05 h^–1^) at 100 °C to TOF = 6000 h^–1^ (*k*_obs_ 49.9 h^–1^) at 140 °C. Depolymerizations were also feasible at higher temperatures but could not be monitored as the kinetics were too fast. The depolymerization activation barrier, attributed to the catalyst-alkoxide backbiting reaction to form cyclohexene oxide, was determined using an Arrhenius analysis. Plots of the logarithm of the observed rate constant (*k*_obs_) against inverse temperature (1/*T*) are linear (Figure 3b). The PCHC depolymerization barrier is 100.2 ± 5.8 kJ mol^–1^, which is higher than the PCHC polymerization activation energy (80.7 kJ mol^–1^) using the same Mg(II)Co(II) catalyst. It is expected that forward polymerization, i.e., CHO + CO_2_, would have a high enthalpic driving force due to release of an epoxide ring strain. It is remarkable that the depolymerization to epoxide has a sufficiently low barrier to be achievable, and the reaction can be favored by removal of the gaseous carbon dioxide and epoxide products.

Other CO_2_-derived polycarbonates were also successfully chemically recycled to epoxides using the Mg(II)Co(II) catalyst. These included poly(cyclopentene carbonate) (PCPC), poly(4-vinylcylcohexene carbonate) (PVCHC), and poly(limonene carbonate) (PLC) (Table 2, entries 1–4, Figure S21). All samples were subjected to the standard depolymerization conditions (catalyst:polycarbonate, 1:300, 140 °C), and in most cases, >80% mass loss occurred in ∼4 min (*k*_obs_ = 49.9–66.6 h^–1^). PLC took significantly longer to depolymerize, with an 80 % mass loss requiring 30 min (*k*_obs_ = 6.9 h^–1^), likely due to the higher boiling point of limonene oxide. All epoxides were isolated after the depolymerization and purity confirmed using ^1^H and ^13^C NMR spectroscopy (Figures S25–S30).

**Table 2:**
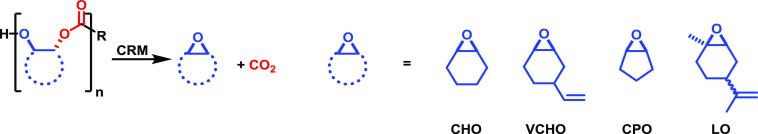
Depolymerization Data for PCHC, PVCHC (poly(vinyl cyclohexene carbonate)), PCPC (poly(cyclopentene carbonate)), and PLC (poly(limonene carbonate)) using Mg(II)Co(II)^a^

entry	polymer	time (s)^b^	TOF (h^–1^)^c^	mass loss rate(kg g^–1^ h^–1^)^d^	*k*_obs_ (h^–1^)^e^	selectivity(%)^f^
1	PCHC	109	6000 (±300)	1.12(±0.06)	49.9(±0.1)	>99
2	PVCHC	88	7300 (±70)	1.63(±0.01)	57.4(±0.05)	>99
3	PCPC	68	9500 (±800)	1.61(±0.1)	66.6(±0.2)	>99
4	PLC	635	1000 (±40)	0.27(±0.01)	6.86(±0.01)	>99

a Polymer films ([Mg(II)Co(II)]:[polycarbonate]_00_ = 1:300) were depolymerized at 140 °C using a N_2_ flow of 25 mL min^–1^ (see SI for further details).

b Interval of time from 20 to 80% mass loss of the polymer.

c TOF = PCHC conversion (20–80%)/moles of catalyst/time (see SI). Error taken from repeat reactions.

d Mass loss rate = mass polycarbonate consumed (20–80% conversion)/catalyst mass/time.

e *k*_obs_ = gradient of linear fits to plots of ln(polycarbonate conversion) vs time (Figures S6 and S22–S24)

f Determined by ^1^H NMR spectroscopy.

**Figure 4:**
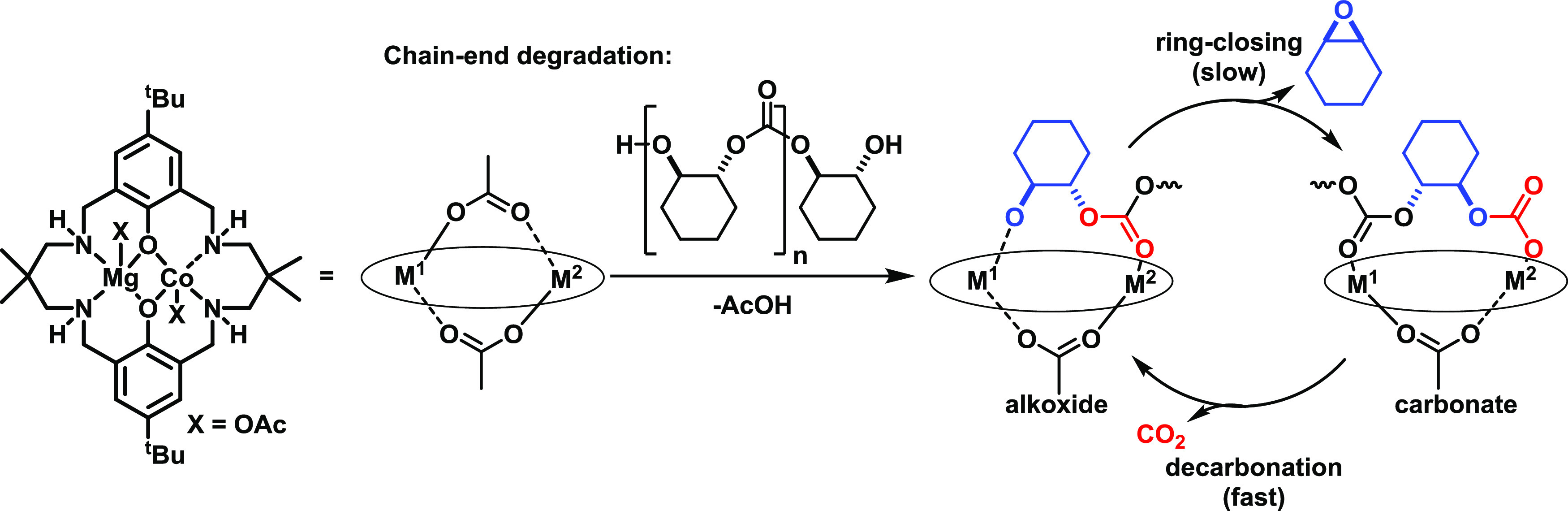
Proposed mechanism for the depolymerization of PCHC catalyzed by Mg(II)Co(II).

To probe the solid-state depolymerization mechanism, samples of cyclic carbonate (*trans*-CHC) were subjected to similar conditions. Thus, films comprising Mg(II)Co(II) and *trans*-CHC (1:300) were analyzed using TGA (Figure S31). Under these reaction conditions, *trans*-CHC also degrades to form CHO and CO_2_, but its rate is an order of magnitude slower than for PCHC (TOF = 800 h^–1^). In the absence of Mg(II)Co(II), a mass loss of *trans-*CHC is also observed, although at a slower rate than when a catalyst is present. Furthermore, in depolymerizations conducted with Mg(II)Co(II) and PCHC (1:300), *trans*-CHC was not detected by ^1^H NMR spectroscopy (Figure S32) or MS (Figure S7) in the crucible or in the evolved gases throughout the reaction. These observations, coupled with the significant rate differences between degradation of PCHC and *trans*-CHC, suggest that *trans*-CHC is not an intermediate in PCHC depolymerization. To understand whether polyethers are relevant intermediates, poly(cyclohexene oxide) (PCHO) was subjected to the same depolymerization conditions (Figure S33). There was no mass loss over 60 min at 140 °C and no depolymerization. This result indicates that the depolymerization mechanism requires a good leaving group in the ring-closing to epoxide step. The poor leaving ability of an alkoxide in comparison to a carbonate may rationalize the failure to depolymerize PCHO. Next, to probe whether depolymerization occurred *via* a chain-scission or chain-end backbiting mechanism, the depolymerization of an acetyl-end capped PCHC (PCHC-OAc, 1:300, solvent cast) was attempted. At 140 °C, no mass loss was observed using PCHC-OAc for up to 2 h (Figure S34). This contrasts with the hydroxyl end-capped PCHC, which under analogous conditions degrades completely in <5 min. Lastly, in a depolymerization conducted at 140 °C with Mg(II)Co(II):PCHC loadings of 1:30, acetic acid was detected in the condensate by ^1^H NMR spectroscopy (Figure S35). The depolymerization mechanism is thus proposed to occur through a PCHC chain backbiting reaction. Accordingly, the PCHC hydroxyl end-groups react with the catalyst to form an alkoxide intermediate, thereby liberating an equivalent of acetic acid. The alkoxide intermediate may then back-bite upon its own chain. This reaction forms an equivalent of epoxide (CHO) and a catalyst-carbonate intermediate. The catalyst-carbonate intermediate rapidly decarboxylates, with carbon dioxide extrusion favored by the moderate gas flow, to (re)form a chain-shortened catalyst-alkoxide intermediate (Figure 4). A related mechanism was proposed for the Mg(II)Mg(II) catalyst.^26^

Finally, the potential to scale-up the solid-state depolymerization was investigated by mixing the catalyst and polymer and heating them under static vacuum with product condensation (Figure S36). The reagents were mixed, using a pestle and mortar and Mg(II)Co(II):PCHC 1:300, heated to 140 °C under a static vacuum (∼10^–2^ mbar) with a collection flask. Using this process, 2 g of PCHC was depolymerized and 1.27 g of CHO was isolated, corresponding to a 92 % isolated yield with the epoxide purity being determined by NMR spectroscopy and boiling point determination (Table S3, entry 1). After the reaction, a fresh quantity of PCHC was added to the residual catalyst and the “second batch” was also successfully depolymerized to CHO, with equivalent selectivity. Further catalyst recycles, up to four in total, were achieved, and in all cases, the catalyst productivity and selectivity for CHO were maintained (Figure S37). MS (MALDI-TOF) and IR spectroscopic analysis of the catalyst residue showed that the complex remained intact post depolymerization (Figures S38 and S39, respectively). These results are encouraging since they demonstrate the feasibility both to scale the reaction and to reuse the catalyst, without requiring any intermediary isolation/purification steps. The larger-scale solid-state depolymerizations were conducted using 2 g of each of the polycarbonates, i.e., PVCHC, PCPC, and PLC, affording the pure epoxides as colorless liquids in up to 95 % isolated yield (Table S3). All these recycled epoxides were used in ROCOP reactions with carbon dioxide to re-form polycarbonates of equivalent molar mass to equivalent samples made using “virgin” epoxide, demonstrating the chemical recycling to monomer concept (Table S4 and Figures S40–43).

## Conclusions

In conclusion, a highly active and selective catalyst, applied in solid-state depolymerizations, for polycarbonate chemical recycling to epoxide and carbon dioxide was reported. The catalytic activity is 26000 h^–1^ or 5 kg polycarbonate/g catalyst/h, and it is tolerant to low loadings, operating efficiently at 0.01 mol %. The depolymerization process was monitored both in situ at a small scale, using TGA, and at a larger scale using 2 g polymer batches. The catalyst can be “recycled” up to four times without compromise to its activity or selectivity. The catalyst and solid state depolymerization methods presented here are expected to be generally useful for other chemical recycling to monomer processes. They open the door to applying CO_2_-derived polymers in sectors where waste polymer recycling would be especially beneficial such as packaging, consumer goods, and automotive sectors, to name a few.

## Supporting Information Available

Experimental methods, characterization, and polymer/monomer purity evaluation data (PDF)

## References

[ref1] Coates G. W.; Getzler Y. D. Y. L. Chemical recycling to monomer for an ideal, circular polymer economy. Nat. Rev. Mater. 2020, 5, 501–516. 10.1038/s41578-020-0190-4.

[ref2] Hong M.; Chen E. Y. X. Chemically recyclable polymers: a circular economy approach to sustainability. Green Chem. 2017, 19, 3692–3706. 10.1039/C7GC01496A.

[ref3] Zhang W.; Dai J.; Wu Y.-C.; Chen J.-X.; Shan S.-Y.; Cai Z.; Zhu J.-B. Highly Reactive Cyclic Carbonates with a Fused Ring toward Functionalizable and Recyclable Polycarbonates. ACS Macro Lett. 2022, 11, 173–178. 10.1021/acsmacrolett.1c00653. 35574765

[ref4] Saxon D. J.; Gormong E. A.; Shah V. M.; Reineke T. M. Rapid Synthesis of Chemically Recyclable Polycarbonates from Renewable Feedstocks. ACS Macro Lett. 2021, 10, 98–103. 10.1021/acsmacrolett.0c00747. 35548994

[ref5] Olsén P.; Odelius K.; Albertsson A.-C. Ring-Closing Depolymerization: A Powerful Tool for Synthesizing the Allyloxy-Functionalized Six-Membered Aliphatic Carbonate Monomer 2-Allyloxymethyl-2-ethyltrimethylene Carbonate. Macromolecules 2014, 47, 6189–6195. 10.1021/ma5012304.

[ref6] Olsén P.; Undin J.; Odelius K.; Keul H.; Albertsson A.-C. Switching from Controlled Ring-Opening Polymerization (cROP) to Controlled Ring-Closing Depolymerization (cRCDP) by Adjusting the Reaction Parameters That Determine the Ceiling Temperature. Biomacromolecules 2016, 17, 3995–4002. 10.1021/acs.biomac.6b01375. 27783494 PMC5155308

[ref7] Schneiderman D. K.; Vanderlaan M. E.; Mannion A. M.; Panthani T. R.; Batiste D. C.; Wang J. Z.; Bates F. S.; Macosko C. W.; Hillmyer M. A. Chemically Recyclable Biobased Polyurethanes. ACS Macro Lett. 2016, 5, 515–518. 10.1021/acsmacrolett.6b00193. 35607243

[ref8] Li C.; Wang L.; Yan Q.; Liu F.; Shen Y.; Li Z. Rapid and Controlled Polymerization of Bio-sourced δ-Caprolactone toward Fully Recyclable Polyesters and Thermoplastic Elastomers. Angew. Chem., Int. Ed. 2022, 61, e202201407 10.1002/anie.202201407. 35150037

[ref9] Zhu J. B.; Watson E. M.; Tang J.; Chen E. Y.-X. A synthetic polymer system with repeatable chemical recyclability. Science 2018, 360, 398–403. 10.1126/science.aar5498. 29700260

[ref10] Hong M.; Chen E. Y. X. Completely recyclable biopolymers with linear and cyclic topologies via ring-opening polymerization of γ-butyrolactone. Nat. Chem. 2016, 8, 42–49. 10.1038/nchem.2391. 26673263

[ref11] Liu Y.; Wu J.; Hu X.; Zhu N.; Guo K. Advances, Challenges, and Opportunities of Poly(γ-butyrolactone)-Based Recyclable Polymers. ACS Macro Lett. 2021, 10, 284–296. 10.1021/acsmacrolett.0c00813. 35570792

[ref12] Tang X.; Hong M.; Falivene L.; Caporaso L.; Cavallo L.; Chen E. Y. X. The Quest for Converting Biorenewable Bifunctional α-Methylene-γ-butyrolactone into Degradable and Recyclable Polyester: Controlling Vinyl-Addition/Ring-Opening/Cross-Linking Pathways. J. Am. Chem. Soc. 2016, 138, 14326–14337. 10.1021/jacs.6b07974. 27700074

[ref13] Zhu J.-B.; Chen E. Y.-X. Living Coordination Polymerization of a Six-Five Bicyclic Lactone to Produce Completely Recyclable Polyester. Angew. Chem., Int. Ed. 2018, 57, 12558–12562. 10.1002/anie.201808003. 30088314

[ref14] Cederholm L.; Wohlert J.; Olsén P.; Hakkarainen M.; Odelius K. “Like Recycles Like:” Selective Ring-Closing Depolymerization of Poly(L-Lactic Acid) to L-Lactide. Angew. Chem., Int. Ed. 2022, 61, e202204531. 10.1002/anie.202204531 PMC9541399 35582840

[ref15] Darensbourg D. J. Comments on the depolymerization of polycarbonates derived from epoxides and carbon dioxide: A mini review. Polym. Degrad. Stab. 2018, 149, 45–51. 10.1016/j.polymdegradstab.2018.01.019.

[ref16] Xu G.; Wang Q. Chemically recyclable polymer materials: polymerization and depolymerization cycles. Green Chem. 2022, 24, 2321–2346. 10.1039/D1GC03901F.

[ref17] Darensbourg D. J.; Wei S.-H.; Yeung A. D.; Ellis W. C. An Efficient Method of Depolymerization of Poly(cyclopentene carbonate) to Its Comonomers: Cyclopentene Oxide and Carbon Dioxide. Macromolecules 2013, 46, 5850–5855. 10.1021/ma401286x.

[ref18] Darensbourg D. J.; Yeung A. D.; Wei S.-H. Base initiated depolymerization of polycarbonates to epoxide and carbon dioxide co-monomers: a computational study. Green Chem. 2013, 15, 1578–1583. 10.1039/c3gc40475g.

[ref19] Liu Y.; Zhou H.; Guo J.-Z.; Ren W.-M.; Lu X.-B. Completely Recyclable Monomers and Polycarbonate: Approach to Sustainable Polymers. Angew. Chem., Int. Ed. 2017, 56, 4862–4866. 10.1002/anie.201701438. 28371275

[ref20] Yu Y.; Fang L.-M.; Liu Y.; Lu X.-B. Chemical Synthesis of CO2-Based Polymers with Enhanced Thermal Stability and Unexpected Recyclability from Biosourced Monomers. ACS Catal. 2021, 11, 8349–8357. 10.1021/acscatal.1c01376.

[ref21] Li C.; Sablong R. J.; van Benthem R. A. T. M.; Koning C. E. Unique Base-Initiated Depolymerization of Limonene-Derived Polycarbonates. ACS Macro Lett. 2017, 6, 684–688. 10.1021/acsmacrolett.7b00310. 35650870

[ref22] Carrodeguas L. P.; Chen T. T. D.; Gregory G. L.; Sulley G. S.; Williams C. K. High elasticity, chemically recyclable, thermoplastics from bio-based monomers: carbon dioxide, limonene oxide and ε-decalactone. Green Chem. 2020, 22, 8298–8307. 10.1039/D0GC02295K.

[ref23] Childers M. I.; Longo J. M.; Van Zee N. J.; LaPointe A. M.; Coates G. W. Stereoselective Epoxide Polymerization and Copolymerization. Chem. Rev. 2014, 114, 8129–8152. 10.1021/cr400725x. 25007101

[ref24] Wang Y.; Darensbourg D. J. Carbon dioxide-based functional polycarbonates: Metal catalyzed copolymerization of CO2 and epoxides. Coord. Chem. Rev. 2018, 372, 85–100. 10.1016/j.ccr.2018.06.004.

[ref25] Scharfenberg M.; Hilf J.; Frey H. Functional Polycarbonates from Carbon Dioxide and Tailored Epoxide Monomers: Degradable Materials and Their Application Potential. Adv. Funct. Mater. 2018, 28, 1704302 10.1002/adfm.201704302.

[ref26] Singer F. N.; Deacy A. C.; McGuire T. M.; Williams C. K.; Buchard A. Chemical Recycling of Poly(Cyclohexene Carbonate) Using a Di-Mg(II) Catalyst. Angew. Chem., Int. Ed. 2022, 61, e2022017 10.1002/anie.202201785. PMC9322669 35442558

[ref27] Yu Y.; Gao B.; Liu Y.; Lu X.-B. Efficient and Selective Chemical Recycling of CO2-based Alicyclic Polycarbonates via Catalytic Pyrolysis. Angew. Chem., Int. Ed. 2022, 61, e202204492. 10.1002/anie.202204492 35770495

[ref28] Koning C.; Wildeson J.; Parton R.; Plum B.; Steeman P.; Darensbourg D. J. Synthesis and physical characterization of poly(cyclohexane carbonate), synthesized from CO2 and cyclohexene oxide. Polymer 2001, 42, 3995–4004. 10.1016/S0032-3861(00)00709-6.

[ref29] Spyridakou M.; Gardiner C.; Papamokos G.; Frey H.; Floudas G. Dynamics of Poly(cyclohexene carbonate) as a Function of Molar Mass. ACS Appl. Polym. Mater. 2022, 4, 3833–3843. 10.1021/acsapm.2c00299.

[ref30] Paul S.; Zhu Y.; Romain C.; Brooks R.; Saini P. K.; Williams C. K. Ring-opening copolymerization (ROCOP): synthesis and properties of polyesters and polycarbonates. Chem. Commun. 2015, 51, 6459–6479. 10.1039/C4CC10113H. 25688813

[ref31] Guerin W.; Diallo A. K.; Kirilov E.; Helou M.; Slawinski M.; Brusson J.-M.; Carpentier J.-F.; Guillaume S. M. Enantiopure Isotactic PCHC Synthesized by Ring-Opening Polymerization of Cyclohexene Carbonate. Macromolecules 2014, 47, 4230–4235. 10.1021/ma5009397.

[ref32] Sulley G. S.; Gregory G. L.; Chen T. T. D.; Peńa Carrodeguas L.; Trott G.; Santmarti A.; Lee K.-Y.; Terrill N. J.; Williams C. K. Switchable Catalysis Improves the Properties of CO2-Derived Polymers: Poly(cyclohexene carbonate-b-ε-decalactone-b-cyclohexene carbonate) Adhesives, Elastomers, and Toughened Plastics. J. Am. Chem. Soc. 2020, 142, 4367–4378. 10.1021/jacs.9b13106. 32078313 PMC7146851

[ref33] Liao X.; Cui F.; He J.-H.; Ren W.-M.; Lu X.-B.; Zhang Y.-T. Sustainable Approach for Synthesis and Completely Recycle of Cyclic CO2-based Polycarbonates. Chem. Sci. 2022, 13, 6283–6290. 10.1039/D2SC01387H. 35733884 PMC9159078

[ref34] Deacy A. C.; Kilpatrick A. F. R.; Regoutz A.; Williams C. K. Understanding metal synergy in heterodinuclear catalysts for the copolymerization of CO2 and epoxides. Nat. Chem. 2020, 12, 372–380. 10.1038/s41557-020-0450-3. 32221501

[ref35] Reis N. V.; Deacy A. C.; Rosetto G.; Durr C. B.; Williams C. K. Heterodinuclear Mg(II)M(II) (M=Cr, Mn, Fe, Co, Ni, Cu and Zn) Complexes for the Ring Opening Copolymerization of Carbon Dioxide/Epoxide and Anhydride/Epoxide. Chem. – Eur. J. 2022, 28, e202104198 10.1002/chem.202104198. 35114048 PMC9306976

