# Supplementary material for: Solid-State Chemical Recycling of Polycarbonates to Epoxides and Carbon Dioxide Using a Heterodinuclear Mg(II)Co(II) Catalyst
Source: J Am Chem Soc. 2022 Sep 28;144(40):18444–9. doi: 10.1021/jacs.2c06937 (PMC9562274; doi:10.1021/jacs.2c06937)
Supplement: Supplementary file 1 — ja2c06937_si_001.pdf [file ja2c06937_si_001.pdf]

Supporting Information:

Solid-State Chemical Recycling of Polycarbonates to Epoxides and Carbon Dioxide using a Heterodinuclear Mg(II)Co(II) Catalyst

Thomas M. McGuire,[a] Arron C. Deacy,[a] Antoine Buchard[b]* and Charlotte K. Williams[a]*

[a] Department of Chemistry, Chemistry Research Laboratory, University of Oxford, 12 Mansfield Rd, Oxford, OX1 3TA, U.K

[b] Department of Chemistry, University of Bath, Centre for Sustainable and Circular Technologies, Claverton Down, Bath BA2 7AY, U.K.

Corresponding Authors

Charlotte K. Williams: Email: charlotte.williams@chem.ox.ac.uk

Antoine Buchard:. Email: a.buchard@bath.ac.uk

Contents

Supporting Information: **1**

Solid-State Chemical Recycling of Polycarbonates to Epoxides and Carbon Dioxide using a Heterodinuclear Mg(II)Co(II) Catalyst **1**

1. Materials 6
2. Methods 8

   2.1 Large scale synthesis of PCHC 8

   2.2.Synthesis of Poly(4-vinylcyclohexene carbonate) (PVCHC) 8

   2.3. Synthesis of Poly(cyclopentene carbonate) (PCPC) 9

   2.4 Synthesis of Poly(limonene carbonate) (PLC) 9

   2.5 Solid-solid Method of Depolymerisation of PCHC 10

   2.6 Solvent-casting Method for the Depolymerisation of Polycarbonates 10

   2.7 Isolation of the Epoxides after Depolymerization 10

   2.8 Depolymerisation of PCHC at Larger-scale 11

   2.9 Repolymerization of Recycled Epoxides 12

3. Synthesis of Polycarbonates 13

   Fig. S1. SEC chromatograms of PCHC (black), PVCHC (red), PCPC (blue), PLC (green) and PPC (mangenta). 14

4. Additional Information 15

   Fig. S2. TGA thermograms showing repeat, independent experiments monitoring PCHC mass loss against time in three PCHC: Mg(II)Co(II) mixtures (300:1, black squares, blue triangles and red circles) 15

   Fig. S3. TGA thermograms showing mass loss against time in PCHC: Mg(II)Co(II) mixtures (300:1) in air (green square) and N_2 (red circles). 15

   Fig. S4. Experimental aparatus used to isolate the epoxides after the depolymerization reactions on the TGA. 16

   Fig. S5. ^{1}H NMR spectrum ($CDCl_3$) of CHO isolated from the depolymerisation of PCHC 17

   Fig. S6. ^{13}C {^{1}H} NMR spectrum ($CDCl_3$) of CHO isolated from the depolymerisation of PCHC. 17

   Fig. S7. Weight % (left axis) and normalized ion intensity (right axis, blue line = 98.1 m/z, red = 44.0 m/z) *vs* time for the depolymerization of PCHC ($[Mg(II)Co(II)]_0$:$[PCHC]_0$ = 1:300) performed on a TGA-MS.. 18

   Fig. S8. Plot of Ln $mass_t/mass_0$ *vs* Time (min) for the depolymerization of PCHC catalysed by Mg(II)Co(II) (Table 1, entry 1, 140 °C, $[PCHC]_0$:$[Mg(II)Co(II)]_0$ 300:1).. 19

   Fig. S9. Plot of Ln $mass_t/mass_0$ *vs* Time (min) for the depolymerization of PCHC catalysed by Mg(II)Co(II) (Table 1, entry 2, 140 °C, $[PCHC]_0$:$[Mg(II)Co(II)]_0$ 300:1).. 19

   Fig. S10. Plot of Ln $mass_t/mass_0$ *vs* Time (min) for the depolymerization of PCHC catalysed by Mg(II)Mg(II) (Table 1, entry 3, 140 °C, $[PCHC]_0$:$[Mg(II)Mg(II)]_0$ 300:1). 20

   Fig. S11. Plot of Ln $mass_t/mass_0$ *vs* Time (min) for the depolymerization of PCHC catalysed by Mg(II)Co(II) (Table 1, entry 4, 140 °C, $[PCHC]_0$:$[Mg(II)Co(II)]_0$ = 1000:1). 20

   Fig. S12. Plot of Ln $mass_t/mass_0$ *vs* Time (min) for the depolymerization of PCHC catalysed by Mg(II)Co(II) (Table 1, entry 5, 140 °C, $[PCHC]_0$:$[Mg(II)Co(II)]_0$ = 2500:1). 21

   Fig. S13. Plot of Ln $mass_t/mass_0$ *vs* Time (min) for the depolymerization of PCHC catalysed by Mg(II)Co(II) (Table 1, entry 6, 140 °C, $[PCHC]_0$:$[Mg(II)Co(II)]_0$ = 5000:1). 22

Fig. S14. Plot of Ln $mass_t/mass_0$ *vs* Time (min) for the depolymerization of PCHC catalysed by Mg(II)Co(II) (Table 1, entry 7, 140 °C, $[PCHC]_0:[Mg(II)Co(II)]_0$ = 10000:1).. 22

Fig. S15. Plot of Ln $mass_t/mass_0$ *vs* Time (min) for the depolymerization of PCHC catalysed by Mg(II)Co(II) (Table 1, entry 8, 100 °C, $[PCHC]_0:[Mg(II)Co(II)]_0$ = 300:1).. 23

Fig. S16. Plot of Ln $mass_t/mass_0$ *vs* Time (min) for the depolymerization of PCHC catalysed by Mg(II)Co(II) (Table 1, entry 9, 110 °C, $[PCHC]_0:[Mg(II)Co(II)]_0$ = 300:1).. 24

Fig. S17. Plot of Ln $mass_t/mass_0$ *vs* Time (min) for the depolymerization of PCHC catalysed by Mg(II)Co(II) (Table 1, entry 10, 120 °C, $[PCHC]_0:[Mg(II)Co(II)]_0$ = 300:1). 25

Fig. S18. Plot of Ln $mass_t/mass_0$ *vs* Time (min) for the depolymerization of PCHC catalysed by Mg(II)Co(II) (Table 1, entry 11, 130 °C, $[PCHC]_0:[Mg(II)Co(II)]_0$ = 300:1).. 25

Fig. S19. Representative TGA thermograms showing mass loss against time in a PCHC: Mg(II)Co(II) mixture (300:1, blue triangles, left axis), PCHC without catalyst (black squares, left axis) and the furnace temperature (red dashed line, right axis). 26

Table S2. Impact of N_2 flow on the depolymerisation of PCHC monitored by TGA 26

Fig. S20. TGA thermograms showing mass loss against time in mixtures of PCHC: Mg(II)Co(II) (300:1, black squares, 1000:1 red circles, 2500:1 green triangles, 5000:1 blue diamonds, 10000:1 purple pentagons).. 27

Fig. S21. TGA thermograms showing mass loss against time in the depolymerization of PCHC (black squares), PVCHC (blue circles), PCPC (red triangles) and PLC (green diamonds).. 28

Fig. S22. Plot of Ln $mass_t/mass_0$ *vs* Time (min) for the depolymerization of PCHC catalysed by Mg(II)Co(II) (Table 2, entry 2, 140 °C, $[PVCHC]_0:[Mg(II)Co(II)]_0$ = 300:1). 29

Fig. S23. Plot of Ln $mass_t/mass_0$ *vs* Time (min) for the depolymerization of PCPC catalysed by Mg(II)Co(II) (Table 2, entry 3, 140 °C, $[PCPC]_0:[Mg(II)Co(II)]_0$ = 300:1).. 29

Fig. S24. Plot of Ln $mass_t/mass_0$ *vs* Time (min) for the depolymerization of PCPC catalysed by Mg(II)Co(II) (Table 2, entry 3, 140 °C, $[PLC]_0:[Mg(II)Co(II)]_0$ = 300:1). 30

Fig. S25. ^{1}H NMR spectrum ($CDCl_3$) of VCHO isolated from the depolymerisation of PVCHC... 31

Fig. S26. ^{13}C {^{1}H} NMR spectrum ($CDCl_3$) of VCHO isolated from the depolymerisation of PVCHC. 31

Fig. S27. ^{1}H NMR spectrum ($CDCl_3$) of CHO isolated from the depolymerisation of PCPC. 32

Fig. S28. ^{13}C {^{1}H} NMR spectrum ($CDCl_3$) of CPO isolated from the depolymerisation of PCPC. 32

Fig. S29. ^{1}H NMR spectrum ($CDCl_3$) of LO isolated from the depolymerisation of PLC. 33

Fig. S30. ^{13}C {^{1}H} NMR spectrum ($CDCl_3$) of LO isolated from the depolymerisation of PCPC. . 33

Fig. S31. TGA thermograms showing mass loss against time in mixtures of PCHC: Mg(II)Co(II) (300:1, blue triangles), *trans*-cyclohexene carbonate: Mg(II)Co(II) (300:1, purple squares) and *trans*-cyclohexene carbonate (red circles). 34

Fig. S32. ^{1}H NMR spectra ($CDCl_3$) of polymer:catalyst residue in TGA crucible for depolymerization at various %mass loss. (N.B. *trans*CHC expected at ⍰ = 3.98 ppm). 35

Fig. S33. TGA thermograms showing mass loss against time in a mixtures of PCHO: Mg(II)Co(II) (300:1, orange triangles) PCHO (blue squares) and the furnace temperature (red dashed line, right axis). 36

Fig. S34. TGA thermograms showing mass loss against time in mixtures of acetyl end-capped PCHC (PCHC-OAc: Mg(II)Co(II) (300:1, orange circles) and PCHC-OAc (black squares) and the furnace temperature (red dashed line, right axis). 37

Fig. S35. ^{1}H NMR spectrum ($CDCl_3$) of CHO formed through Mg(II)Co(II) catalyzed depolymerization of PCHC (1:30, 140 °C).. 38

Fig. S36. Vacuum transfer apparatus. From left to right: flask continaing polymer and catalyst, T-junction and collection flask 39

Fig. S37. Productivity of the catalyst over 4 reaction cycles. 40

Fig. S38. MS (MALDI-TOF) spectrum of catalyst before (purple) and after (black) depolymerization 40
Fig. S39. IR spectra of Mg(II)Co(II) before (purple) and after (black) depolymerization. 41
Table S3. Depolymerisation of polycarbonates to isolate epoxides......42
Table S4. Polymerisation of epoxides formed through chemical recycling......42
Fig. S40. SEC trace of original (black) and recycled (red) PCHC......43
Fig. S41. SEC trace of original (black) and recycled (red) PVCHC. 43
Fig. S42. SEC trace of original (black) and recycled (red) PCPC......44
Fig. S43. SEC trace of original (black) and recycled (red) PLC......44
5. NMR Characterisation of Polycarbonates 45
Fig. S44. ^{1}H NMR spectrum ($CDCl_3$) of PCHC. 45
Fig. S45. ^{13}C {^{1}H} NMR spectrum ($CDCl_3$) of PCHC......45
Fig. S46. ^{1}H NMR spectrum ($CDCl_3$) of PVCHC......46
Fig. S47. ^{13}C {^{1}H} NMR spectrum ($CDCl_3$) of PVCHC......46
Fig. S48. ^{1}H NMR spectrum ($CDCl_3$) of PCPC......47
Fig. S49. ^{13}C {^{1}H} NMR spectrum ($CDCl_3$) of PCPC......47
Fig. S50. ^{1}H NMR spectrum ($CDCl_3$) of PLC......48
Fig. S51. ^{13}C {^{1}H} NMR spectrum ($CDCl_3$) of PLC. 48
8. References 49

1. Materials

All experiments were carried out under N_2 using standard Schlenk/glovebox techniques unless otherwise stated. Epoxides were purchased from commercial sources (Acros organics, Sigma Aldrich, alfa aesar) and used as received unless otherwise stated. All solvents used were anhydrous unless otherwise stated. THF and toluene were obtained from an SPS system, degassed by several freeze-pump-thaw cycles and stored over 3 Å molecular sieves under nitrogen. Mesitylene was dried over CaH_2 and vacuum transferred before storing over 3 Å molecular sieves under nitrogen. 1,2-*trans*-cyclohexenediol (Sigma Aldrich) was recrystallised from anhydrous ethyl acetate. Research-grade carbon dioxide was dried through a Drierite column and two additional drying columns (Micro Torr, Model number: MC1-804FV) in series before use. The pro-ligand,[1] Mg(II)Mg(II),[2] Mg(II)Co(II),[3] Co(III)K(I) [4] and Al(III)Tris[5] were synthesised according to literature procedures.

Size exclusion chromatography (SEC) was carried out on a Shimadzu LC-20AD instrument using two PSS SDV linear M columns in series, with a THF eluent. Measurements were conducted at 30 °C with a flow rate of 1 mL/min. Samples were detected with a differential refractive index (RI) detector. Number-average molar mass ($M_{n,SEC}$),and dispersities, ($Đ_M$ (M_w/M_n)) were calculated against a polystyrene calibration. The polymer samples were dissolved in HPLC-grade THF at a concentration of *ca* 10 mg/mL and filtered through a 0.2 0μm microfilter prior to analysis

Differential scanning calorimetry (DSC) was performed using a TA discovery 25 auto. Experiments were performed under N_2 flow (50 mL/min) using aluminium TZERO pans. Samples (2–5 mg) were equilibrated at 20 °C then cooled to 0 °C at a rate of 20 °C/min and held at 0 °C for 5 minutes. The sample was then heated at a rate of 20 °C/min to 200 °C and held at 200 °C for 5 minutes. The sample was then cooled at a rate of 20 °C/min to and held at 0 °C for 5 minutes. The heating and cooling cycles were repeated three times and thermal data is reported from the second heating cycle.

Thermal gravimetric analysis (TGA) was performed on a TGA/DSC 1 system (Mettler-Toledo Ltd). Details of depolymerization experiments conducted on the TGA are given in the methods section.

NMR spectra were obtained using a Bruker AVIII HD nanobay NMR spectrometer. Coupling constants are given in Hertz. Selectivities were determined by ^{1}H NMR spectroscopy.

MALDI-TOF MS spectra were recorded on a Bruker MALDI-MS Autoflex. The Matrix was trans-2-[3-(4-tert-butylphenyl)-2-methyl-2- propenylidene]-malonitrile. The samples were prepared by mixing 10 mg/mL THF solutions of complex and matrix together in a 1:4 ratio. The mixture (2 μL) was spotted on a MALDI-plate and left to dry under an inert atmosphere. Spectra were recorded in positive reflector mode with laser intensity and gain varied.

Turnover Frequency (TOF) calculations were performed using mass loss against time plots from 20-80% mass loss of the polymer over time. For solvent-cast samples, the mass loss of the solvent was taken into account by running blank samples (i.e. polymer samples which had been solvent cast without catalyst present). The full TOF caluclations, with adjustments for the presence of solvent, are included as a separate excel spreadsheet. An example calculation is shown below:

Solvent-cast depolymerisation: cat:polymer 1:300 (Table 1, entry 2)

Mass loss due to residual solvent (taken from blank) = 9%

Therefore, polymer mass accounts for 91% mass of the sample

Corrected mass at 20% conversion of the sample = 91%- 0.2*91% = 72.8%

Corrected mass at 80% conversion of the sample = 91%-0.8*91% = 18.2%

From %mass vs time plot, time to 72.8% mass = 1.76 minutes

From %mass vs time plot, time to 18.2% mass = 3.57 minutes

TOF= (mmol of polymer converted/mmol of catalyst)/time

60% mass loss = 60% mmol of polymer converted.

TOF = 0.6/(1/300) / ((3.57-1.76/60))

= 6000 h^{-1}

2. Methods

2.1 Large scale synthesis of PCHC

Under an atmosphere of air, cyclohexene oxide (CHO, 48.5 g, 494.14 mmol, 10000.00 equiv.) was added to an autoclave reactor containing 1,2-*trans*-cyclohexene diol (CHD, 0.29 g, 2.47 mmol, 50.00 equiv.) and Co(III)K(I) (31 mg, 0.0494, 1.00 equiv.). The reactor was pressurised to 30 bar CO_2 with stirring. Once the pressure stabilised, the reactor was heated to 50 °C. After 24 h, the pressure had dropped to 1 bar. The autoclave was opened and the reaction product dissolved in DCM. Acetic acid (*ca* 2 mL) was added to the polymer solution and the product was precipitated from methanol. The supernatant was decanted and the precipitation was repeated until there was no colouration of the polymer (5 times). The residue was dried, under vacuum, to yield PCHC as a white powder (44.4 g, 63% yield).

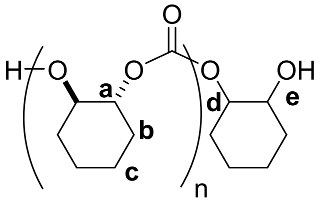

^{1}H NMR (400 MHz, $CDCl_3$) δ 4.82 – 4.51 (m, 2H, H_a), 4.49 – 4.32 (m, *end group,* H_d), 3.70 – 3.48 (m, *end group*, H_e), 2.25 – 1.93 (m, 2H, H_b), 1.85 – 1.61 (m, 2H, H_c), 1.60 – 0.98 (m, 4H, H_b, H_c).

^{13}C NMR (151 MHz, $CDCl_3$) δ 157.4 – 150.6 (**C=O**), 84.3 – 81.0 (C_d), 77.9 – 77.5 (C_a), 73.3 – 71.4 (C_e), 31.1 – 27.4 (C_b), 25.0 – 21.1 (C_c).

$M_{n,SEC}$ = 8000 (1.07), T_g = 122 °C $T_{d,onset}$ = 255 °C

2.2.Synthesis of Poly(4-vinylcyclohexene carbonate) (PVCHC)

PVCHC was synthesised and isolated using an analogous method to PCHC (28.2 g, 44% yield)

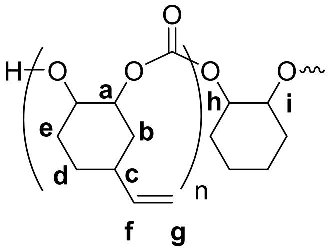

^{1}H NMR (400 MHz, $CDCl_3$) δ 5.85 – 5.66 (m, 1H, H_f), 5.15 – 4.96 (m, 2H, H_g), 4.93 – 4.85 (m, 1H, H_a), 4.82 – 4.73 (m, 1H, H_a), 4.57 – 4.50 (m, *end group*, H_h), 3.94 – 3.86 (m, *end group*, H_i), 3.79 – 3.71 (m, H_i) 2.55 – 2.37 (m, 1H, H_c), 1.99 –1.59 (m, 5H, H_b, H_d, H_e).

^{13}C NMR (151 MHz, $CDCl_3$) δ 154.3 – 152.8 (**C=O**), 142.5 – 140.8 (C_f), 114.9 – 113.3 (C_g), 78.4 – 77.7 (C_h), 74.8 – 72.5 (C_a), 68.3 – 67.5 (C_i), 35.5 – 34.5 (C_c), 32.4 – 31.1 (C_e), 26.67 – 25.70 (C_b, C_d),

$M_{n,SEC}$ = 10200 (1.08), T_g = 112 °C $T_{d,onset}$ = 282 °C

2.3. Synthesis of Poly(cyclopentene carbonate) (PCPC)

Under an atmosphere of air, cyclopentene oxide (CPO, 12.5 g, 148.60 mmol, 1000.00 equiv.) was added to an autoclave reactor containing 1,2-*trans*-cyclohexene diol (CHD, 0.346 g, 2.98 mmol, 20.00 equiv.) and Co(III)K(I) (93 mg, 0.149, 1.00 equiv.). The reactor was pressurised to 30 bar CO_2 with stirring. Once the pressure stabilised, the reactor was heated to 50 °C. After 72 h, the pressure had dropped to 17 bar. The autoclave was opened and the reaction product dissolved in DCM. Acetic acid (*ca* 2 mL) was added to the polymer solution and the product was precipitated from methanol. The supernatant was decanted, the residue redissolved and the polymer solution was flushed through a silica plug using an eluent of DCM to DCM:acetone (1–5% acetone). The solvent was removed under vacuum to yield PCPC as a white powder (11.1 g, 58% yield).

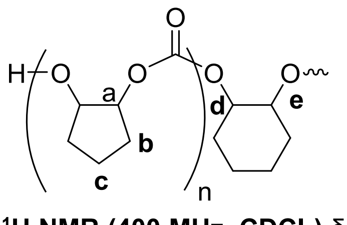

^{1}H NMR (400 MHz, $CDCl_3$) δ 5.10 – 4.88 (m, 2H, H_a), 4.79 – 4.68 (m, *end group,* H_d), 4.22 – 4.08 (m, *end group*, H_e), 2.23 – 1.94 (m, 2H, $\boldsymbol{H_b}$), 1.89 – 1.58 (m, 4H, $\boldsymbol{H_b}$, $\boldsymbol{H_c}$).

^{13}C NMR (151 MHz, $CDCl_3$) δ 155.1 – 153.1 (**C=O**), 87.1 – 86.5 ($\boldsymbol{C_a}$), 82.8 – 81.7 ($\boldsymbol{C_d}$), 78.1 – 77.5 ($\boldsymbol{C_e}$), 30.4 – 29.7 ($\boldsymbol{C_b}$), 21.5 – 21.0 ($\boldsymbol{C_c}$).

$\boldsymbol{M_{n,SEC}}$ = 4100 (1.06), $\boldsymbol{T_g}$ = 72 °C $\boldsymbol{T_{d,onset}}$ = 259 °C

2.4 Synthesis of Poly(limonene carbonate) (PLC)

Under an atmosphere of air, limonene oxide (LO, 10.0 g, 65.69 mmol, 346.00 equiv.) was added to an autoclave reactor containing bis(triphenylphosphoranylidene)ammonium chloride (PPNCl, 0.11 g, 0.19 mmol, 0.50 equiv.) and Al(III)Tris (0.194 g, 0.38 mmol, 1.00 equiv.). The vessel was pressurised to 15 bar CO_2 with stirring. Once the pressure stabilised, the reaction was heated to 45 °C. After 72 h, the pressure had dropped to 5 bar. The autoclave was opened and the reaction product dissolved in DCM. Acetic acid (*ca* 1 mL) was added to the polymer solution and the product was precipitated from methanol. The residue was redissolved and flushed through a silica plug using an eluent of DCM to DCM:acetone (1–5% acetone). The solvent was removed under vacuum to yield PLC as a white powder (11.1 g, 58% yield).

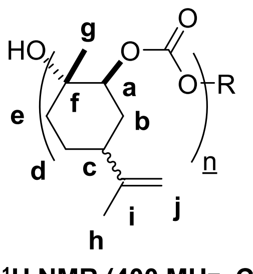

^{1}H NMR (400 MHz, $CDCl_3$) δ 5.31 – 4.95 (m, 1H, $\boldsymbol{H_a}$), 4.70 (s, 2H, $\boldsymbol{H_j}$), 2.66 – 2.08 (m, 2H, $\boldsymbol{H_c}$, $\boldsymbol{H_e}$), 2.02 – 1.15 (m, 11 H, $\boldsymbol{H_b}$, $\boldsymbol{H_d}$, $\boldsymbol{H_e}$, $\boldsymbol{H_f}$, $\boldsymbol{H_g}$). *Chlroine end group not observed*

^{13}C NMR (151 MHz, $CDCl_3$) δ 154.4 – 150.5 (***C=O***), 149.42 – 148.43 ($\boldsymbol{C_i}$), 110.3 – 108.6 ($\boldsymbol{C_j}$), 82.7 – 80.9 ($\boldsymbol{C_f}$), 76.8 – 74.8 ($\boldsymbol{C_a}$), 38.6 – 36.7 ($\boldsymbol{C_c}$), 32.0 – 30.3 ($\boldsymbol{C_b}$**,** $\boldsymbol{C_e}$), 26.4 – 25.5 ($\boldsymbol{C_d}$), 22.2 – 21.5 ($\boldsymbol{C_h}$), 21.5 – 20.4 ($\boldsymbol{C_g}$).

$\boldsymbol{M_{n,SEC}}$ = 7700 (1.26), $\boldsymbol{T_g}$ = 219 °C $\boldsymbol{T_{d,onset}}$ = 97 °C

2.5 Solid-solid Method of Depolymerisation of PCHC

In the glovebox, PCHC (426 mg, 3.00 mmol) and Mg(II)Co(II) (7.5 mg, 0.01 mmol) were added to a pestle and mortar. The mixture was ground to a fine powder and transferred to an aluminium Tzero TGA crucible. The crucible was crimped in the glovebox with a hermetic seal. The crucible was then transferred to the TGA instrument for monitored solid-state depolymerisation. The following TGA method template was used for the reaction:

N_2 flow of 25.0 mL min^{-1}
Jump to 140 °C
Isotherm for 60 minutes
Ramp at 20 °C min^{-1} to 500 °C
Jump to 30 °C.

The N_2 flow rate, reaction temperature and length of the isotherm were varied as required.

2.6 Solvent-casting Method for the Depolymerisation of Polycarbonates

In the glovebox, PCHC (142 mg, 1.00 mmol) was added to a vial and dissolved in THF (1 mL). Mg(II)Co(II) was added to the vial as a stock solution (0.01 M in THF). The catalyst:polymer stock-solution (40 µL) was transferred to an aluminium Tzero TGA crucible. The crucible was placed under vacuum for 30 minutes before being crimped in the glovebox with a hermetic seal. The crucible was then transferred to a TGA instrument for monitored solid-state depolymerisation using the method outlined in section 2.5.

2.7 Isolation of the Epoxides after Depolymerization

A depolymerization experiment was setup as in section 2.5 or 2.6.

A pre-weighed 2-necked round-bottomed flask was attached to the outlet of the TGA instrument. The 2-necked round-bottomed flask was cooled over N_2(l)/isopropanol mixture. The following TGA method template was used for the reaction:

N_2 flow of 25.0 mL min^{-1}
Equilibrate at 30 °C
Jump to 140 °C
Isotherm for 60 minutes
Jump to 30 °C.
N_2 flow of 100.0 mL min^{-1}
Isotherm for 10 minutes

The weight of the flask was recorded post-run to calculate yields for depolymerisation and ^{1}H NMR spectroscopy was used to confirm the product selectivity.

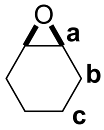

^{1}H NMR (400 MHz, $CDCl_3$) δ 3.10 (dq, J = 2.3, 1.1 Hz, 2H, H_a), 1.99 – 1.87 (m, 2H, H_b), 1.86 – 1.67 (m, 2H, H_b), 1.49 – 1.34 (m, 2H, H_c), 1.29 – 1.14 (m, 2H, H_c).

^{13}C NMR (151 MHz, $CDCl_3$) δ 52.2 (C_a), 24.5 (C_b), 19.5 (C_c)

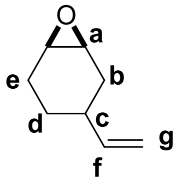

^{1}H NMR (400 MHz, $CDCl_3$) δ 5.70 (tdd, J = 17.1, 10.4, 6.6 Hz, 1H, H_f), 5.09 – 4.84 (m, 2H, H_g), 3.28 – 3.08 (m, 2H, H_a), 2.29 – 1.07 (m, H_b, H_c, H_d, H_e).

^{13}C NMR (151 MHz, $CDCl_3$) δ 143.1 (C_g), 143.0 (C_g), 113.0 (C_f), 112.9 (C_f), 52.9 (C_a), 52.4 (C_a), 51.6 (C_a), 51.4 (C_a), 36.9 (C_c), 33.6 (C_c), 30.9 (C_b), 30.1 (C_b), 26.6 (C_d), 25.1 (C_e), 24.3 (C_d), 23.3 (C_e).

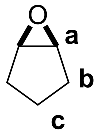

^{1}H NMR (400 MHz, $CDCl_3$) δ 3.45 (s, 1H, H_a), 2.06 – 1.92 (m, 2H, H_b), 1.62 – 1.47 (m, 2H, H_b, H_c), 1.43 – 1.24 (m, 1H, H_c)

^{13}C NMR (151 MHz, $CDCl_3$) δ 57.2 (C_a), 27.2 (C_b), 18.3 (C_c).

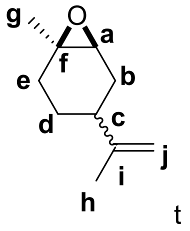

^{1}H NMR (400 MHz, $CDCl_3$) δ 4.72 – 4.66 (m, 2H, H_j), 3.04 – 2.99 (m, 1H, H_a), 2.19 —1.09 (m, H_b, H_c, H_d, H_e, H_g, H_h).

^{13}C NMR (151 MHz, $CDCl_3$) δ 149.30 (C_i), 149.10 (C_i), 109.2 (C_j), 109.1 (C_j), 60.6 (C_a), 59.36 (C_a), 57.6 (C_f), 57.4 (C_f), 40.9 (C_c), 36.3 (C_c), 30.9 (C_e), 30.8 (C_b), 30.0 (C_b), 28.7 (C_d), 26.0 (C_e), 24.5 (C_g), 24.4 (C_d), 23.2 (C_g), 21.2 (C_h), 20.3 (C_h).

2.8 Depolymerisation of PCHC at Larger-scale

Under an N_2 atmosphere, PCHC (2.00 g, 14.07 mmol, 300.00 equiv.) was added to an ampoule containing Mg(II)Co(II) (35.2 mg, 0.47 mmol, 1.00 equiv.). The reaction vessel was attached to a glass vacuum-transfer apparatus and placed under dynamic vacuum (~10^{-2} mbar) for 1 h. After 1 h, the apparatus was placed under static vacuum and the reaction vessel was heated to 140 °C for 2 hours, after which atmospheric pressure was re-stablished in the system using N_2. The distillate was then subjected to three freeze-pump-thaw cycles to yield CHO as a clear oil (1.27 g, 92% yield).

2.9 Repolymerization of Recycled Epoxides

The repolymerization of the recycled epoxides was generally performed using slightly different methods to those used to form the virgin polymers owing to the smaller-scales. Nonetheless, control experiments using the virgin epoxide, under identical polymerization conditions, were also carried out to ensure that both the recycled and virgin epoxide gave equivalent activity and selectivity values.

2.9.1 Repolymerisation of Recycled CHO and VCHO

In the glovebox, CHO (679 mg, 6.92 mmol, 1000.00 equiv.) was added to an ampoule containing Mg(II)Co(II) (5.2 mg, 0.00692 mmol, 1.00 equiv.), CHD (4.0 mg, 0.0346 mmol, 5 equiv.) and mesitylene (30 µL, 0.216 mmol, 31.16 equiv. The mixture was dissolved in toluene (1.70 mL) to form a pink solution. On a CO_2-fed Schlenk line, the atmosphere of the reaction flask was exchanged for CO_2. The solution was heated to 100 °C for 3 h before being quenched by cooling to room temperature. The crude reaction mixture was analysed by ^1H NMR spectroscopy and SEC.

2.9.2 Repolymerization of Recycled CPO

In the glovebox, CPO (2.18 g, 25.9, 2000.00 equiv.), was added to a vial containing Mg(II)Co(II) (9.8 mg, 0.0130 mmol, 1.00 equiv.) and CHD (15.4 mg, 0.130 mmol, 10.00 equiv.). The mixture was dissolved in toluene (3.2 mL) to form a pink solution which was transferred to an autoclave. The vessel was sealed and pressurised to 20 bar CO_2 with stirring. Once the pressure stabilised, the reaction was heated to 100 °C. After 92 h, the autoclave was cooled and the reaction product dissolved in DCM. The crude reaction mixture was analysed by ^1H NMR spectroscopy and SEC.

2.9.3 Repolymerization of Recycled LO

In the glovebox, LO (3.50 mL, 22.99 mmol, 100.00 equiv.) was added to a vial contain Al(III)Tris (119 mg, 0.230 mmol, 1.00 equiv.) and PPNCl (66 mg, 0.115 mmol, 0.5 equiv.). The mixture was dissolved in toluene (2.5 mL) to form colourless solution which was transferred to an autoclave. The vessel was sealed and pressurised to 15 bar CO_2 with stirring. Once the pressure stabilised, the reaction vessel was heated to 45 °C. After 116 h, the autoclave was cooled and the reaction product in DCM. The crude reaction mixture was analysed by ^1H NMR spectroscopy and SEC.

3. Synthesis of Polycarbonates

Table S1. Synthesis of Polycarbonates

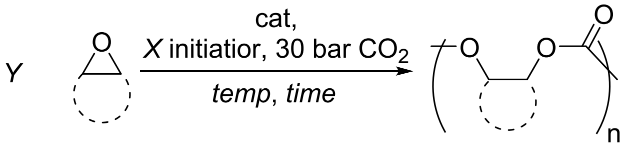

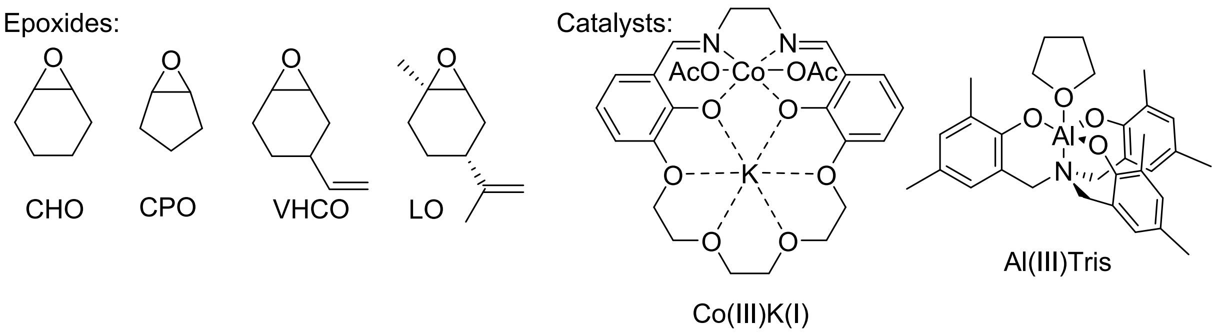

Entry	m	Cat	i	$[m]_0$:$[i]_0$:$[cat]_0$	Time (h)	Temp. (°C)	Conv. %[a]	$M_{n,SEC}$ [$Đ_M$] (g mol^{-1})[c]	T_d [T_g] (°C)[d]
1	CHO	Co(III)K(I)	CHD	10000:50:1	24	50	78%	8000 [1.07]	255 [122]
2	VCHO	Co(III)K(I)	CHD	10000:20:1	20	50	70%	10200 [1.08]	282 [112]
3	CPO	Co(III)K(I)	CHD	1000:20:1	72	50	97%	4100 [1.06]	259 [72]
4	LO	Al(III)Tris	PPNCl	350:1:2	72	45	48%	7700 [1.76]	219 [97]

Reactions performed in neat epoxide using either a 25 mL or 100 mL autoclave. All entries report >99% CO_2 selectivity *vs* theoretical maximum. CHO: cyclohexene oxide, vCHO: 4-vinyl-1,2-cyclohexene oxide, CPO: cyclopentene oxide, LO: limonene oxide.[a] Determined by ^1H NMR spectroscopy ($CDCl_3$) by relative integration of epoxide vs polycarbonate using an internal standard of mesitylene. [c] Calculated by SEC relative to polystyrene standards in tetrahydrofuran (THF) eluent; $Đ_M=M_w/M_n$. [d] T_d = degradation onset measured under N_2, T_g = glass transition reported from second heating cycles in DSC.

. .

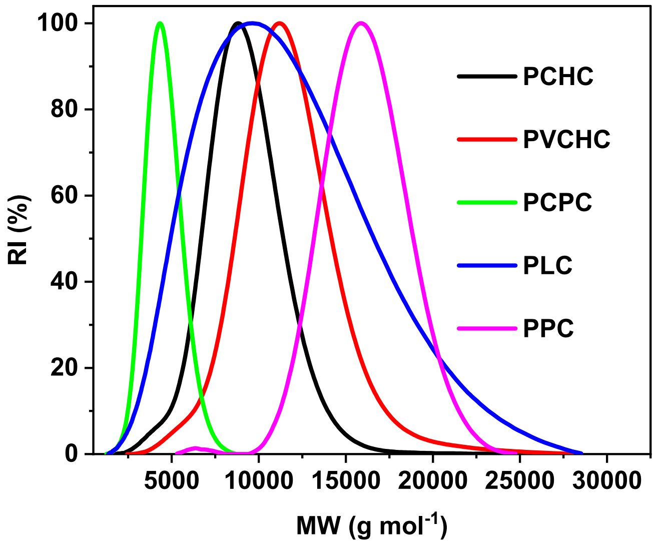

Fig. S1. SEC chromatograms of PCHC (black), PVCHC (red), PCPC (blue), PLC (green) and PPC (mangenta).

4. Additional Information

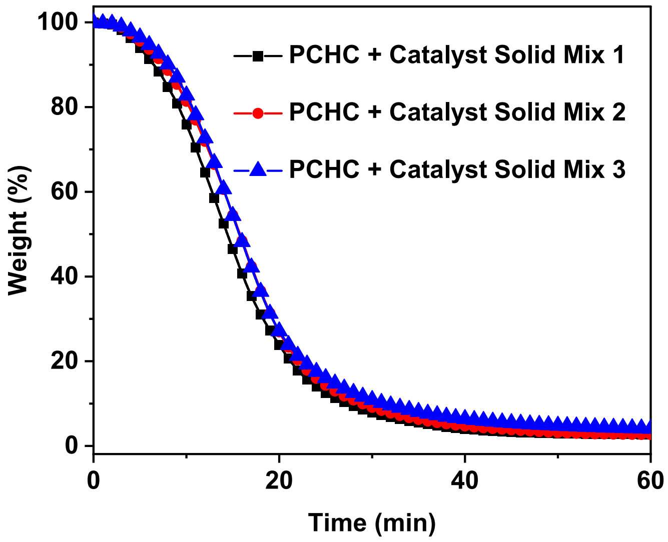

.

Fig. S2. TGA thermograms showing repeat, independent experiments monitoring PCHC mass loss against time in three PCHC: Mg(II)Co(II) mixtures (300:1, black squares, blue triangles and red circles). The samples were prepared using a pestle and mortar. The TGAs were run with an isotherm of 60 minutes at 140 °C.

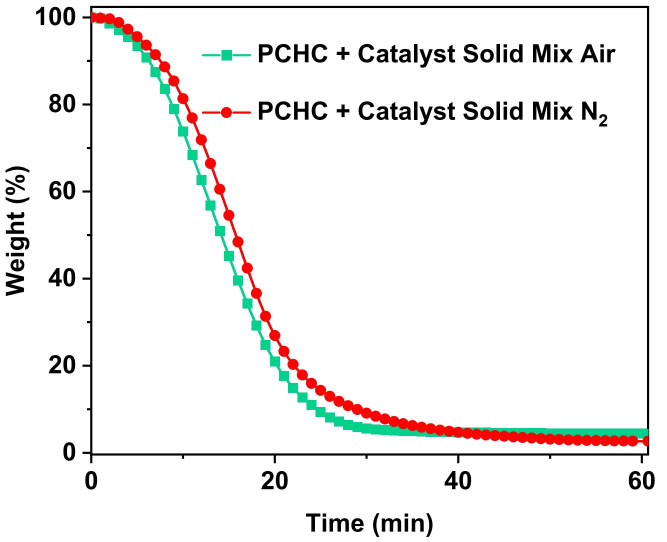

Fig. S3. TGA thermograms showing mass loss against time in PCHC: Mg(II)Co(II) mixtures (300:1) in air (green square) and N_2 (red circles). The samples were prepared by grinding in a pestle and mortar. The TGAs were run with an isotherm of 60 minutes at 140 °C.

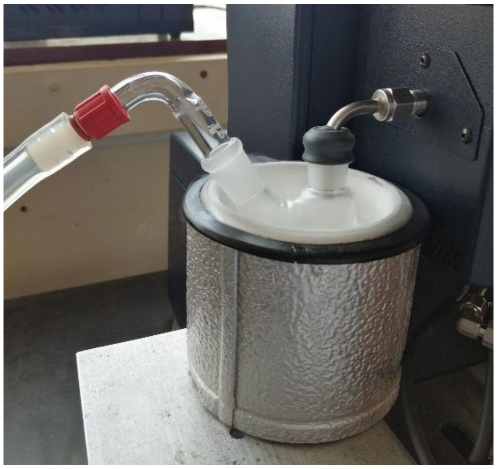

Fig. S4. Experimental aparatus used to isolate the epoxides after the depolymerization reactions on the TGA.

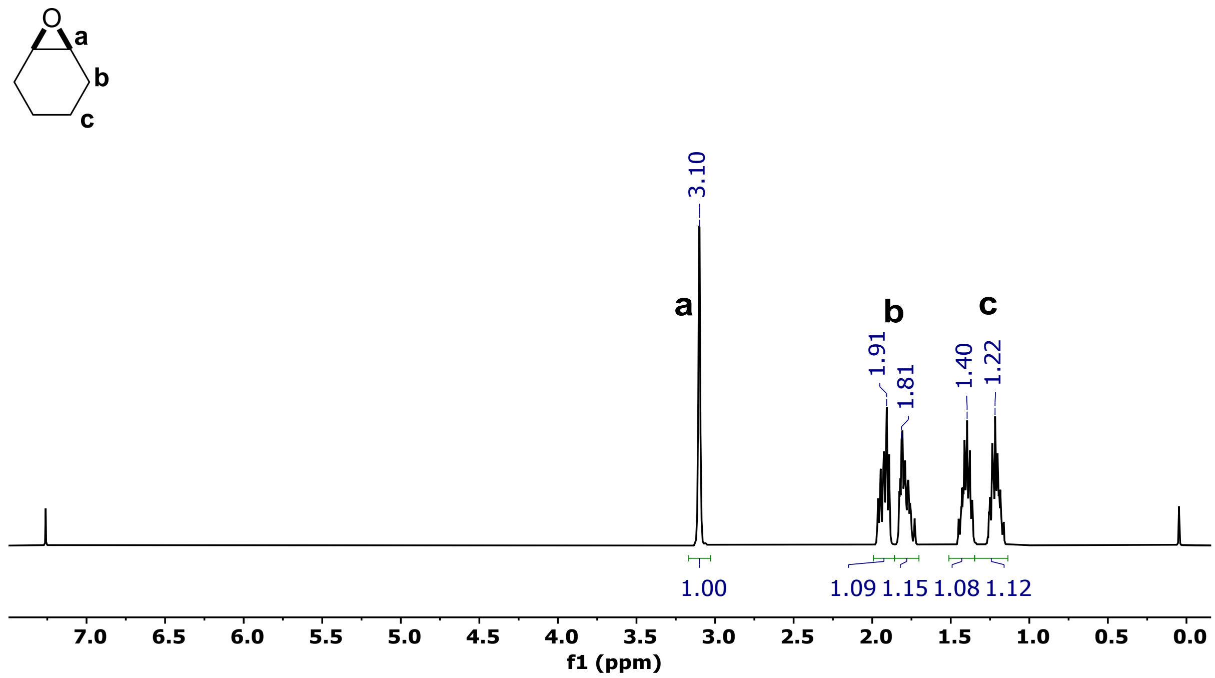

Fig. S5. ^{1}H NMR spectrum ($CDCl_3$) of CHO isolated from the depolymerisation of PCHC.

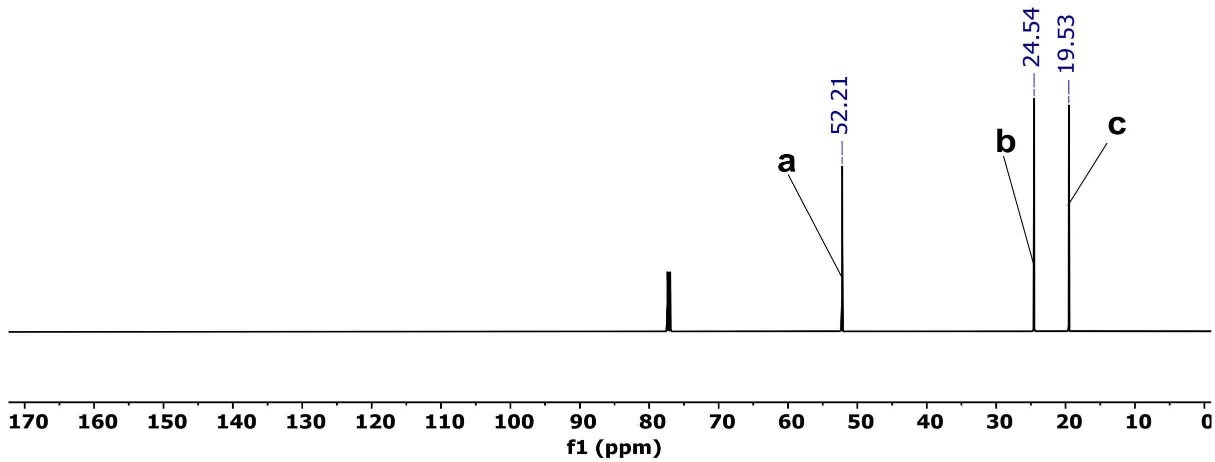

Fig. S6. ^{13}C {^{1}H} NMR spectrum ($CDCl_3$) of CHO isolated from the depolymerisation of PCHC.

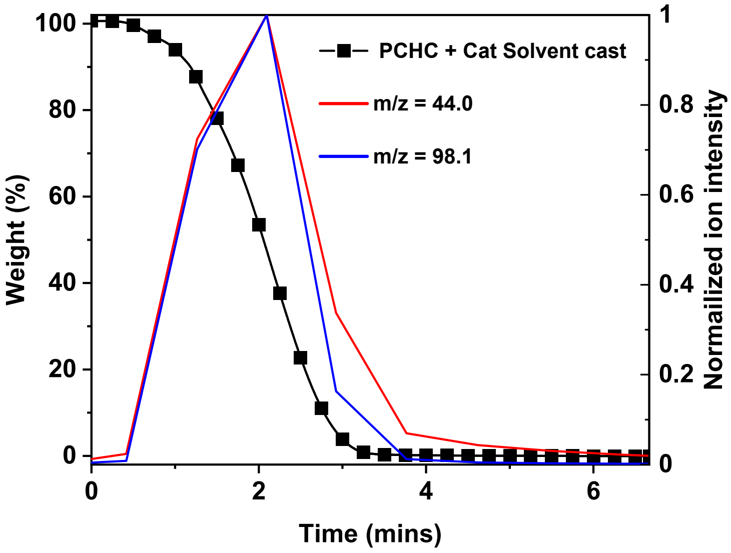

Fig. S7. Weight % (left axis) and normalized ion intensity (right axis, blue line = 98.1 m/z, red = 44.0 m/z) *vs* time data for the depolymerization of PCHC ($[Mg(II)Co(II)]_0$:$[PCHC]_0$ = 1:300) performed on a TGA-MS. The ions with m/z = 98.1 and 44.0 are assigned as CHO^+ (M_r = 98.13 g mol^{-1}) and CO_2^+ (M_r = 44.01 g mol^{-1}), respectively.

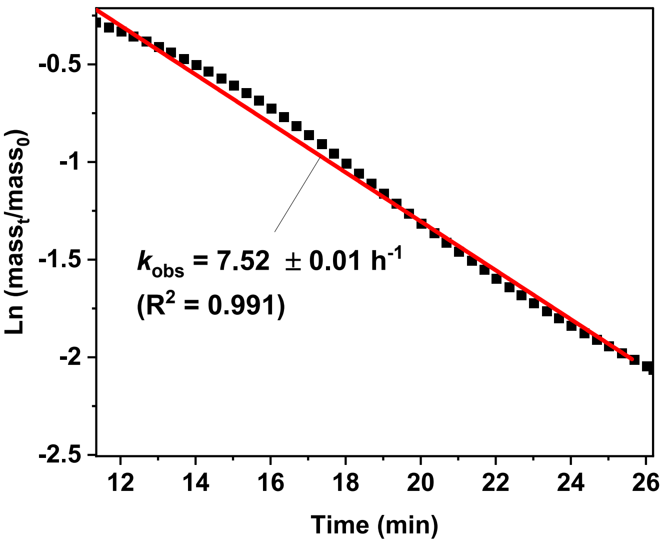

Fig. S8. Plot of Ln mass$_t$/mass$_0$ *vs* Time (min) for the depolymerization of PCHC catalysed by Mg(II)Co(II) (Table 1, entry 1, 140 °C, $[PCHC]_0$:$[Mg(II)Co(II)]_0$ 300:1). The k_{obs} fit was taken from 15 – 82% of mass loss of the polymer. The reaction was prepared by mixing the reagents with a pestle and mortar.

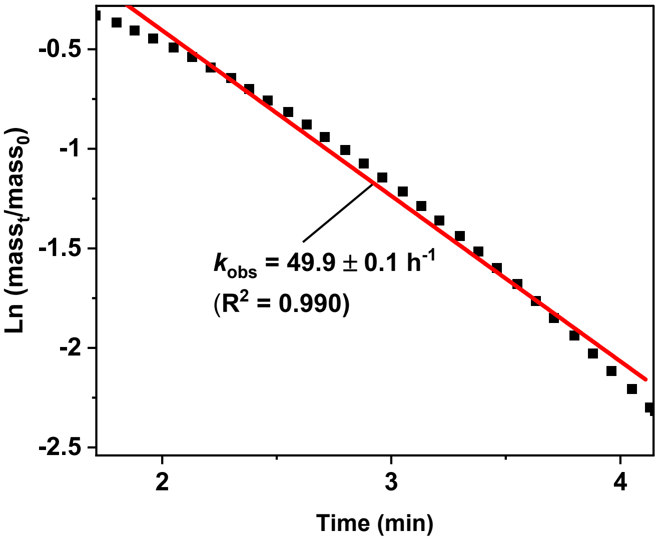

Fig. S9. Plot of Ln mass$_t$/mass$_0$ *vs* Time (min) for the depolymerization of PCHC catalysed by Mg(II)Co(II) (Table 1, entry 2, 140 °C, $[PCHC]_0$:$[Mg(II)Co(II)]_0$ 300:1). The k_{obs} fit was taken from 15 – 82% of mass loss of the polymer. The reaction was prepared by solvent-casting the reagents.

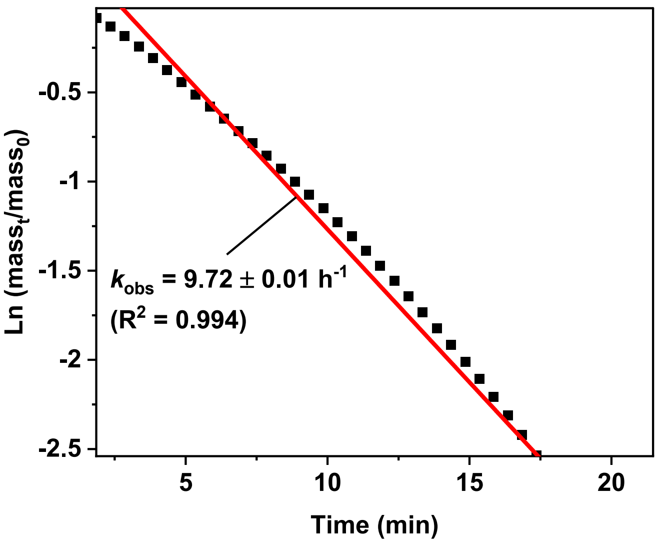

Fig. S10. Plot of Ln $mass_t/mass_0$ *vs* Time (min) for the depolymerization of PCHC catalysed by Mg(II)Mg(II) (Table 1, entry 3, 140 °C, $[PCHC]_0$:$[Mg(II)Mg(II)]_0$ 300:1). The k_{obs} fit was taken from 15 – 82% of mass loss of the polymer. The reaction was prepared by solvent-casting the reagents.

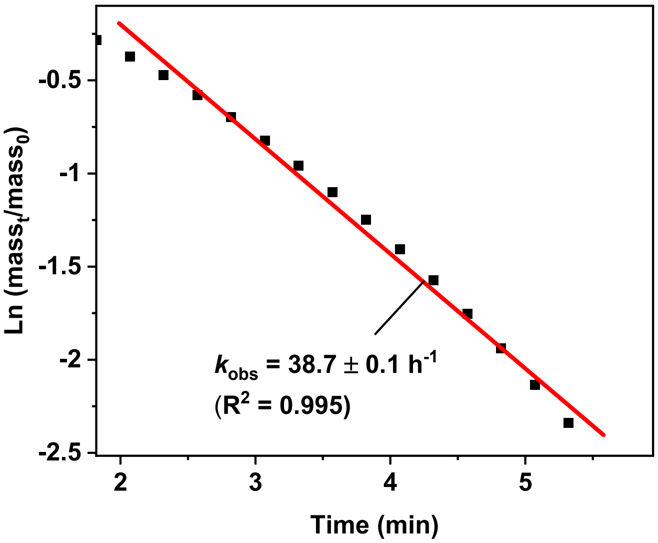

Fig. S11. Plot of Ln $mass_t/mass_0$ *vs* Time (min) for the depolymerization of PCHC catalysed by Mg(II)Co(II) (Table 1, entry 4, 140 °C, $[PCHC]_0$:$[Mg(II)Co(II)]_0$ = 1000:1). The k_{obs} fit was taken from 15 – 82% of mass loss of the polymer.The reaction was prepared by solvent-casting the reagents.

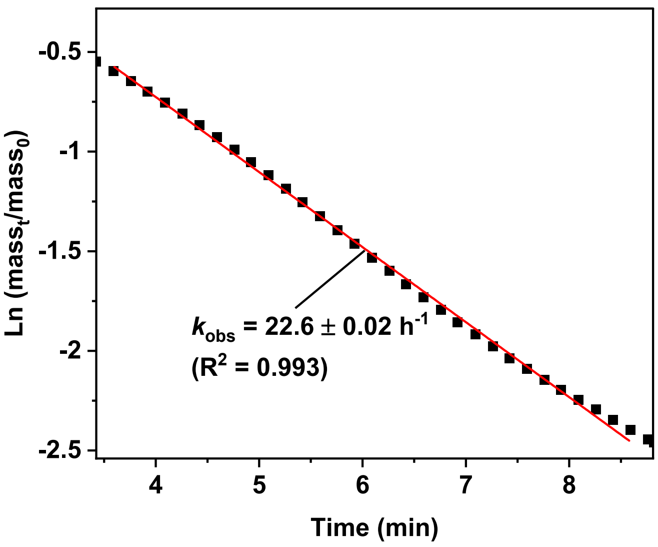

Fig. S12. Plot of Ln $mass_t/mass_0$ *vs* Time (min) for the depolymerization of PCHC catalysed by Mg(II)Co(II) (Table 1, entry 5, 140 °C, $[PCHC]_0$:$[Mg(II)Co(II)]_0$ = 2500:1). The k_{obs} fit was taken from 15 – 82% of mass loss of the polymer. The reaction was prepared by solvent-casting the reagents.

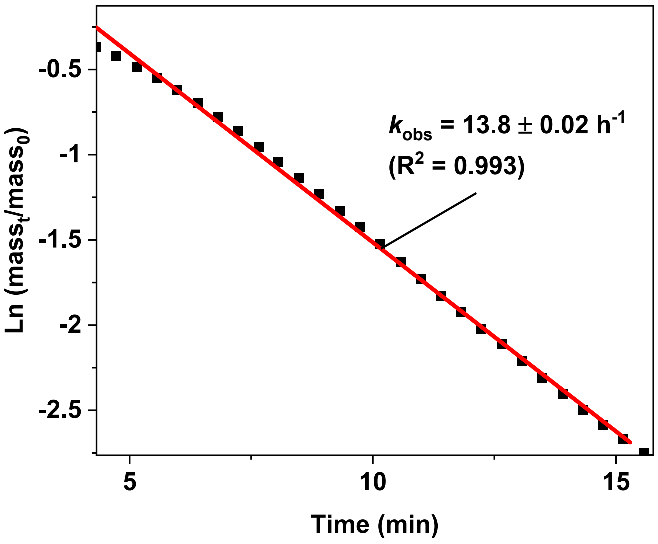

Fig. S13. Plot of Ln $mass_t/mass_0$ *vs* Time (min) for the depolymerization of PCHC catalysed by Mg(II)Co(II) (Table 1, entry 6, 140 °C, $[PCHC]_0$:$[Mg(II)Co(II)]_0$ = 5000:1). The k_{obs} fit was taken from 15 – 82% of mass loss of the polymer. The reaction was prepared by solvent-casting the reagents.

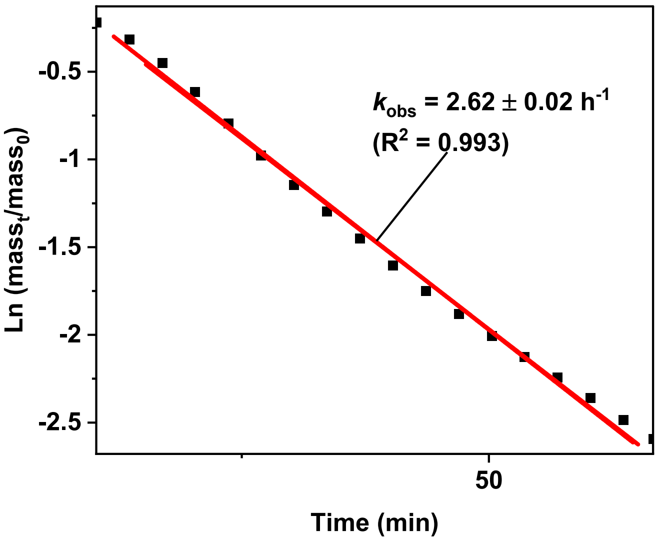

Fig. S14. Plot of Ln $mass_t/mass_0$ *vs* Time (min) for the depolymerization of PCHC catalysed by Mg(II)Co(II) (Table 1, entry 7, 140 °C, $[PCHC]_0$:$[Mg(II)Co(II)]_0$ = 10000:1). The k_{obs} fit was taken from 15 – 82% of mass loss of the polymer. The reaction was prepared by solvent-casting the reagents.

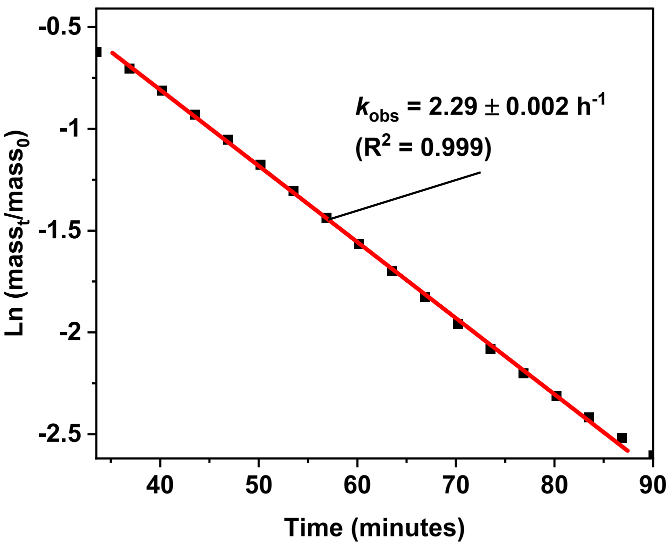

Fig. S15. Plot of Ln mass$_t$/mass$_0$ *vs* Time (min) for the depolymerization of PCHC catalysed by Mg(II)Co(II) (Table 1, entry 8, 100 °C, $[PCHC]_0$:$[Mg(II)Co(II)]_0$ = 300:1). The k_{obs} fit was taken from 35 – 82% of mass loss of the polymer (N.B. different fitting used due to shape of degradation profile). The reaction was prepared by solvent-casting the reagents.

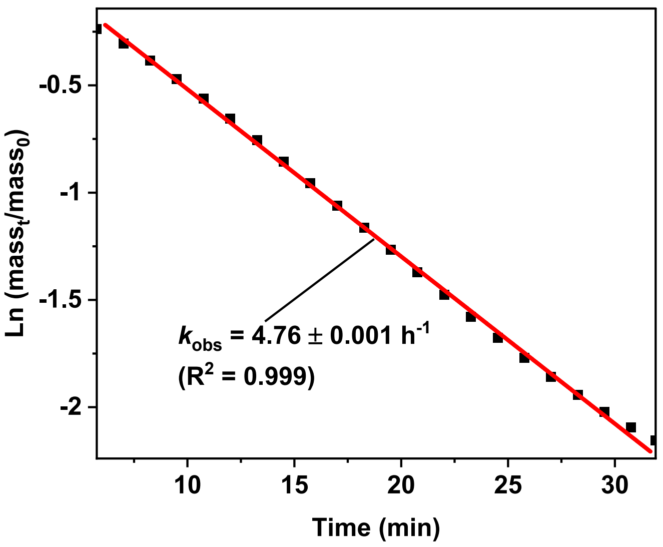

Fig. S16. Plot of Ln $mass_t/mass_0$ *vs* Time (min) for the depolymerization of PCHC catalysed by Mg(II)Co(II) (Table 1, entry 9, 110 °C, $[PCHC]_0$:$[Mg(II)Co(II)]_0$ = 300:1). The k_{obs} fit was taken from 30 – 82% of mass loss of the polymer (N.B. different fitting used due to shape of degradation profile). The reaction was prepared by solvent-casting the reagents.

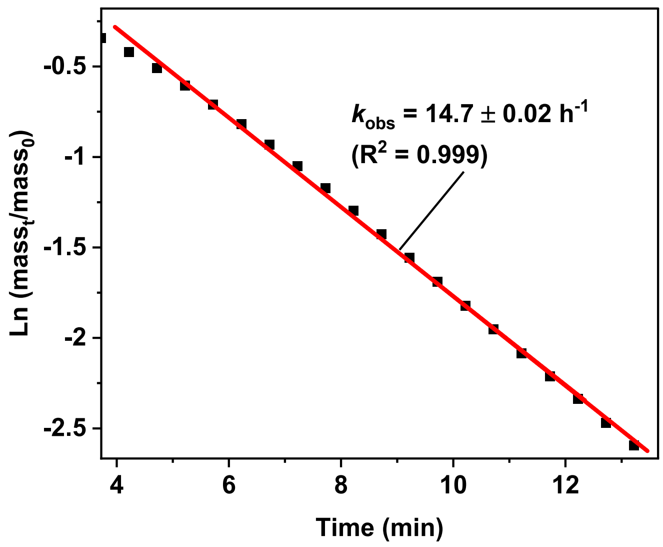

Fig. S17. Plot of Ln mass$_t$/mass$_0$ *vs* Time (min) for the depolymerization of PCHC catalysed by Mg(II)Co(II) (Table 1, entry 10, 120 °C, $[PCHC]_0$:$[Mg(II)Co(II)]_0$ = 300:1). The k_{obs} fit was taken from 15 – 82% of mass loss of the polymer. The reaction was prepared by solvent-casting the reagents.

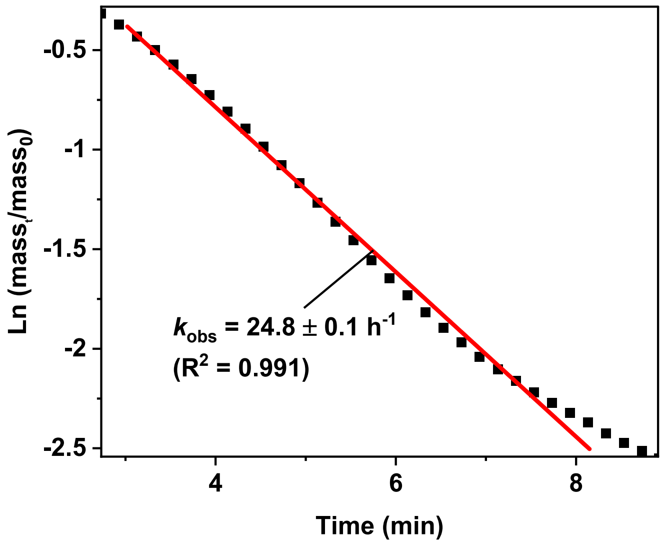

Fig. S18. Plot of Ln mass$_t$/mass$_0$ *vs* Time (min) for the depolymerization of PCHC catalysed by Mg(II)Co(II) (Table 1, entry 11, 130 °C, $[PCHC]_0$:$[Mg(II)Co(II)]_0$ = 300:1). The k_{obs} fit was taken from 15 – 82% of mass loss of the polymer. The reaction was prepared by solvent-casting the reagents.

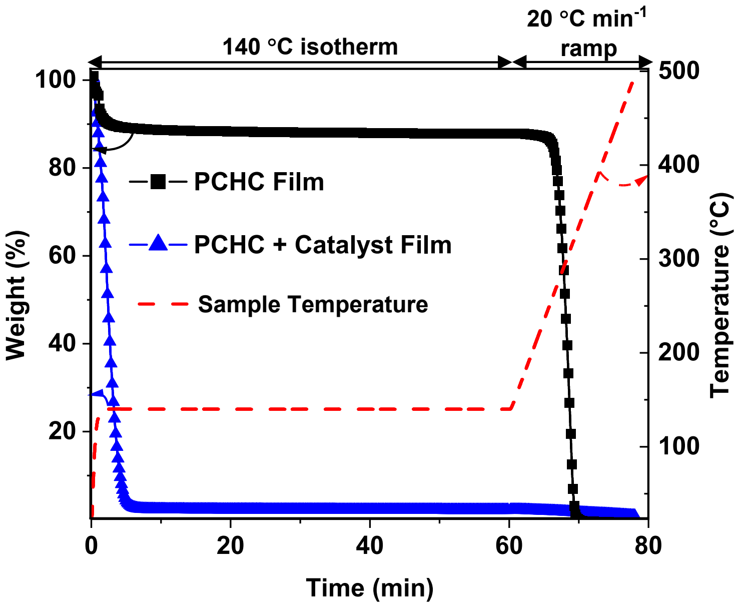

Fig. S19. Representative TGA thermograms showing mass loss against time in a PCHC: Mg(II)Co(II) mixture (300:1, blue triangles, left axis), PCHC without catalyst (black squares, left axis) and the furnace temperature (red dashed line, right axis). The samples were prepared by solvent casting. The TGAs were run with an isotherm of 60 minutes at 140 °C followed by a 20 °C minute^{-1} ramp from 140 °C to 500 °C.

Table S2. Impact of N_2 flow on the depolymerisation of PCHC monitored by TGA

Entry	N_2 flow	Time (s)[b]	k_{obs} (h^{-1})[c]	TOF (h^{-1})[d]	Residual mass[e]
1	12.5	220	48.5	6000	2%
2	25.0	210	49.9	6000	2%
3	50.0	200	49.8	6400	2%
4	100.0	190	51.1	6500	2%
5	200.0	235	42.1	5200	2%
6	350.0	600	14.3	1700	2%
7	500.0	414	17.3	2500	2%

Reactions prepared by solvent casting polymer:catalyst solutions ($[PCHC]_0:[2]_0$ = 300:1, $[PCHC]_0$ = 1.16 M solution in THF) in TGA crucibles and placing under high vacuum for 30 mins. Reactions performed with varied N_2 flow on TGA using the following method: 1) jump from 30 to 140 °C 3) 60 minute isotherm at 140 °C 4) ramp at 20 °C/min to 500 °C.[b] Time taken for reaction to reach 80% mass loss.[c] k_{obs} = gradient of linear fitting of the logarithm of %polymer mass vs time taken from *in-situ* TGA.[d] TOF = moles of PCHC consumed/time*moles of catalyst at 80% mass loss.[e] %mass taken at end of 140 °C isotherm residual mass.

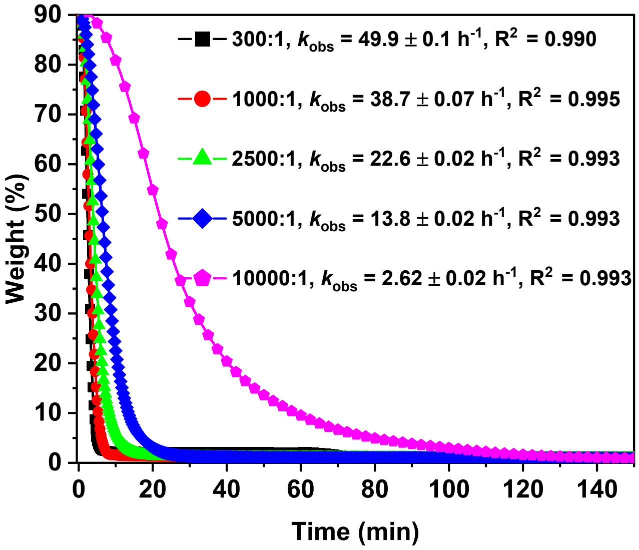

Fig. S20. TGA thermograms showing mass loss against time in mixtures of PCHC: Mg(II)Co(II) (300:1, black squares, 1000:1 red circles, 2500:1 green triangles, 5000:1 blue diamonds, 10000:1 purple pentagons). The samples were prepared by solvent casting. The TGAs were run with an 60–180 minutes isotherm at 140 °C.

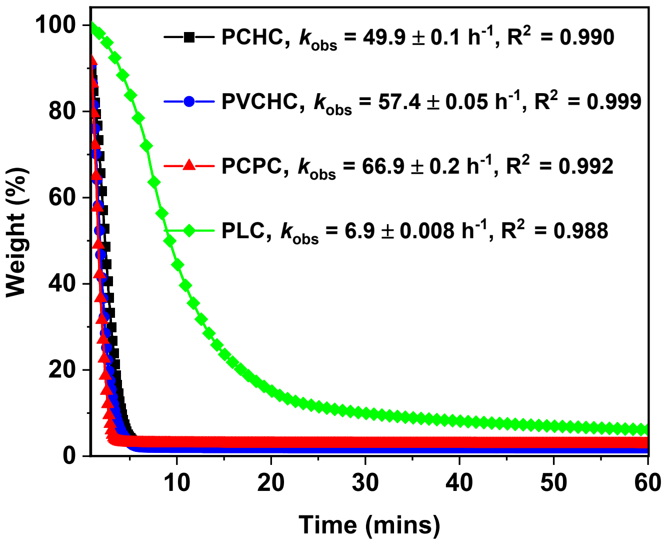

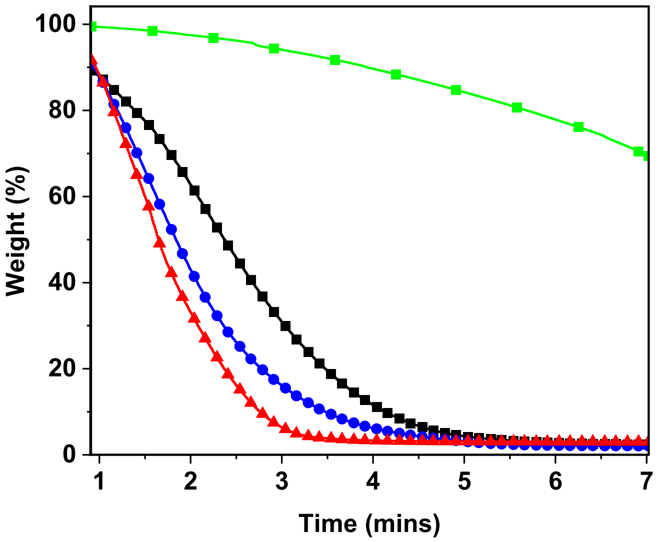

Fig. S21. TGA thermograms showing mass loss against time in the depolymerization of PCHC (black squares), PVCHC (blue circles), PCPC (red triangles) and PLC (green diamonds). The reactions were prepared by solvent casting $[\text{polymer}]_0$:$[\text{Mg(II)Co(II)}]_0$ mixtures (300:1). The top trace shows the thermograms from 0 to 60 minutes and bottom trace shows a zoomed in section to highlight the faster degradations. The TGAs were run with an isotherm of 60 minutes at 140 °C followed by a 20 °C minute^{-1} ramp from 140 °C to 500 °C. All depolymerisations exceeded 94% weight loss following the 140 °C isotherm.

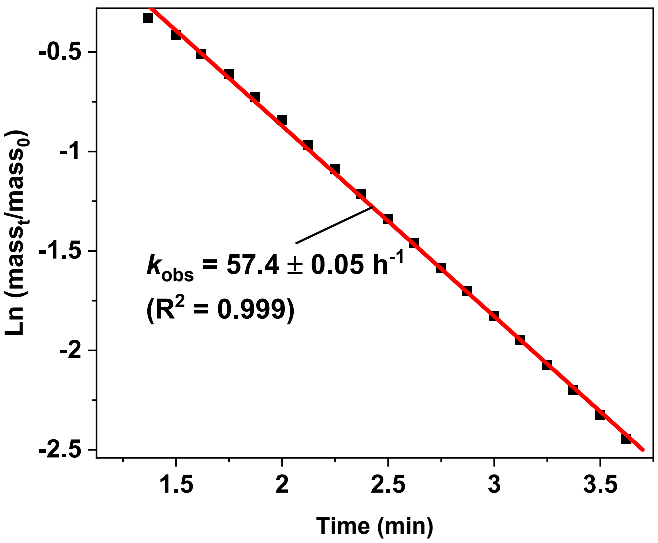

Fig. S22. Plot of Ln $mass_t/mass_0$ *vs* Time (min) for the depolymerization of PCHC catalysed by Mg(II)Co(II) (Table 2, entry 2, 140 °C, $[PVCHC]_0$:$[Mg(II)Co(II)]_0$ = 300:1). The k_{obs} fit was taken from 15 – 82% of mass loss of the polymer. The reaction was prepared by solvent-casting the reagents.

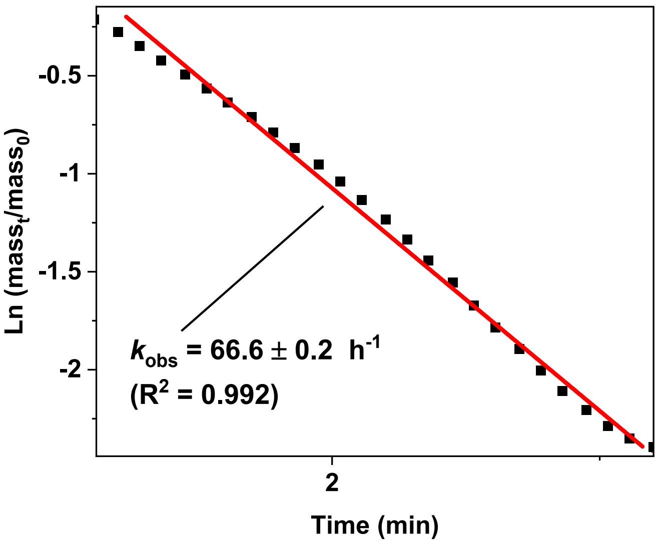

Fig. S23. Plot of Ln $mass_t/mass_0$ *vs* Time (min) for the depolymerization of PCPC catalysed by Mg(II)Co(II) (Table 2, entry 3, 140 °C, $[PCPC]_0$:$[Mg(II)Co(II)]_0$ = 300:1). The k_{obs} fit was taken from 15 – 82% of mass loss of the polymer. The reaction was prepared by solvent-casting the reagents.

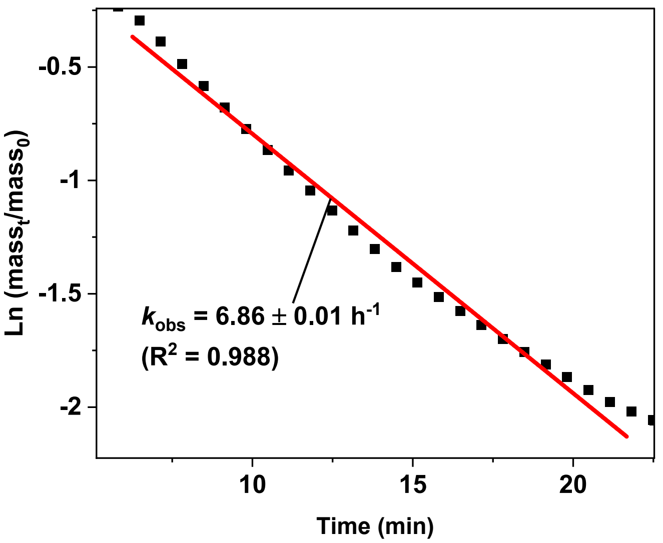

Fig. S24. Plot of Ln $mass_t/mass_0$ *vs* Time (min) for the depolymerization of PCPC catalysed by Mg(II)Co(II) (Table 2, entry 3, 140 °C, $[PLC]_0$:$[Mg(II)Co(II)]_0$ = 300:1). The k_{obs} fit was taken from 15 – 73% of mass loss of the polymer (N.B. different fitting used for this reaction due to shape of degradation profile). The reaction was prepared by solvent-casting the reagents.

.

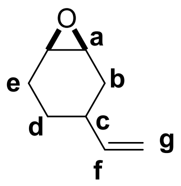

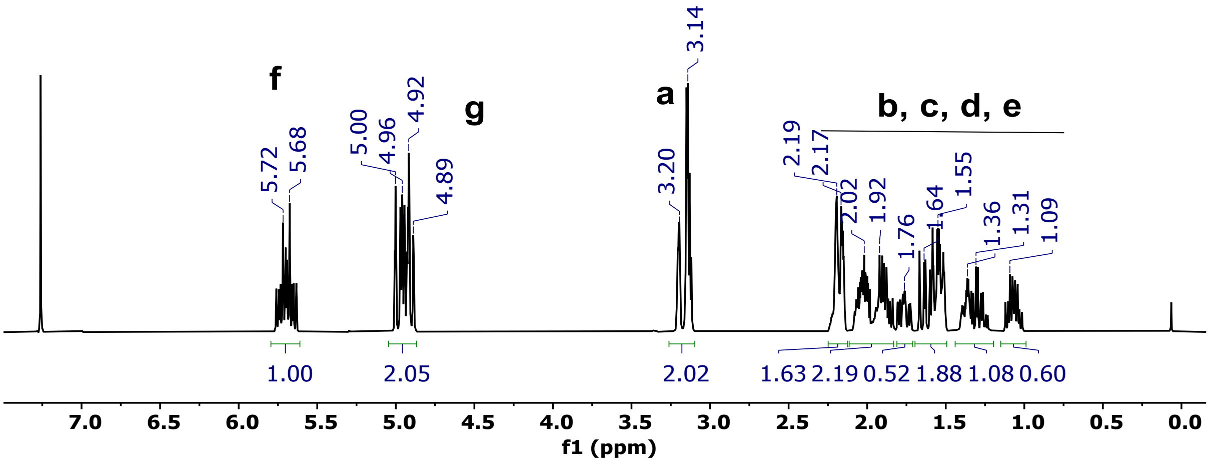

Fig. S25. ^{1}H NMR spectrum ($CDCl_3$) of VCHO isolated from the depolymerisation of PVCHC.

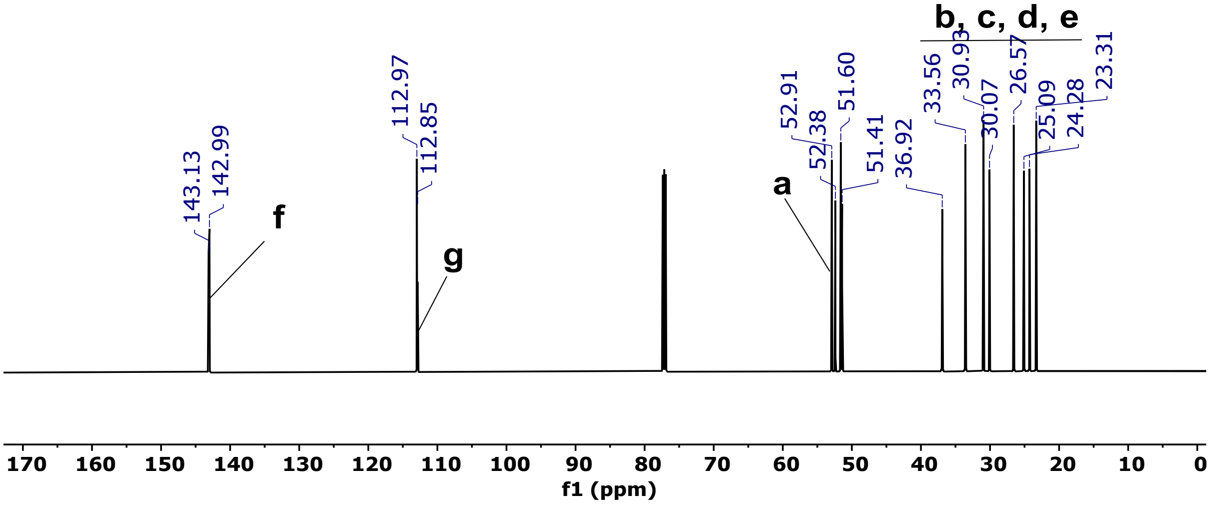

Fig. S26. ^{13}C {^{1}H} NMR spectrum ($CDCl_3$) of VCHO isolated from the depolymerisation of PVCHC.

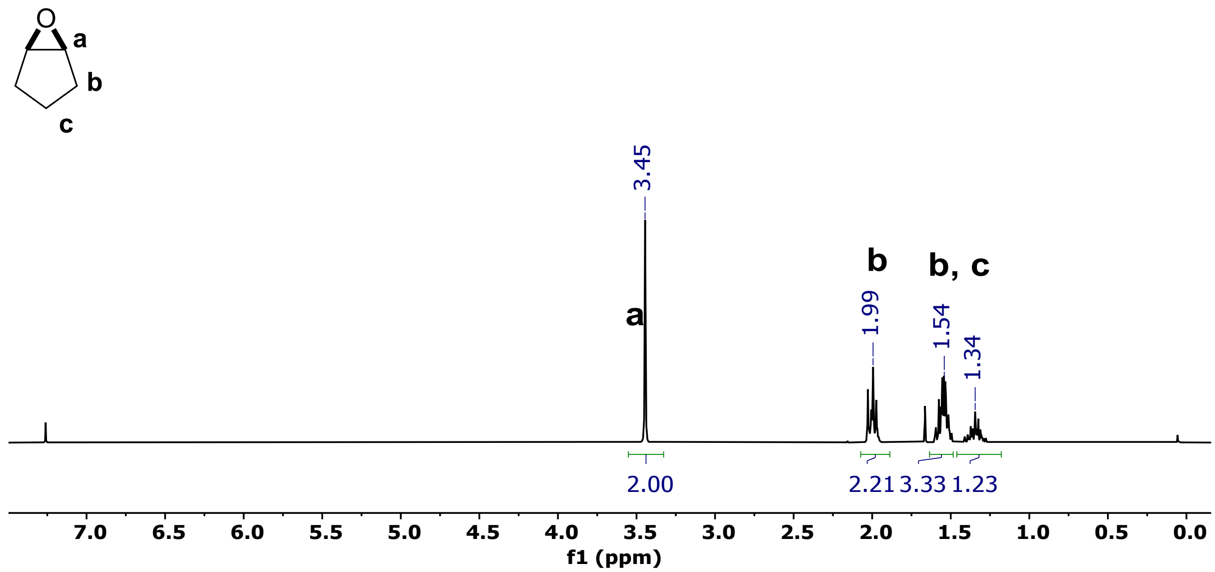

Fig. S27. ^{1}H NMR spectrum ($CDCl_3$) of CHO isolated from the depolymerisation of PCPC.

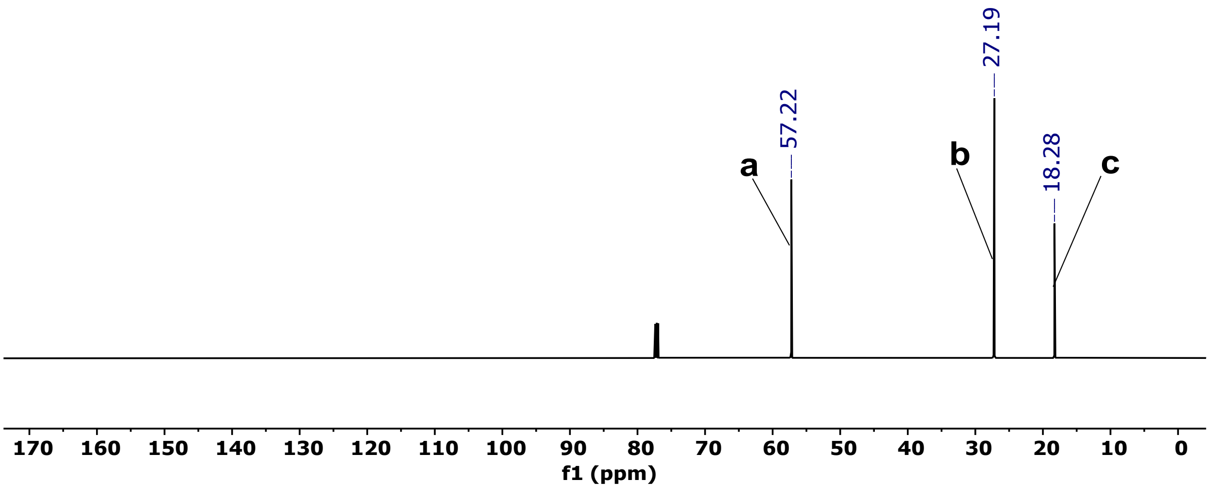

Fig. S28. ^{13}C {^{1}H} NMR spectrum ($CDCl_3$) of CPO isolated from the depolymerisation of PCPC.

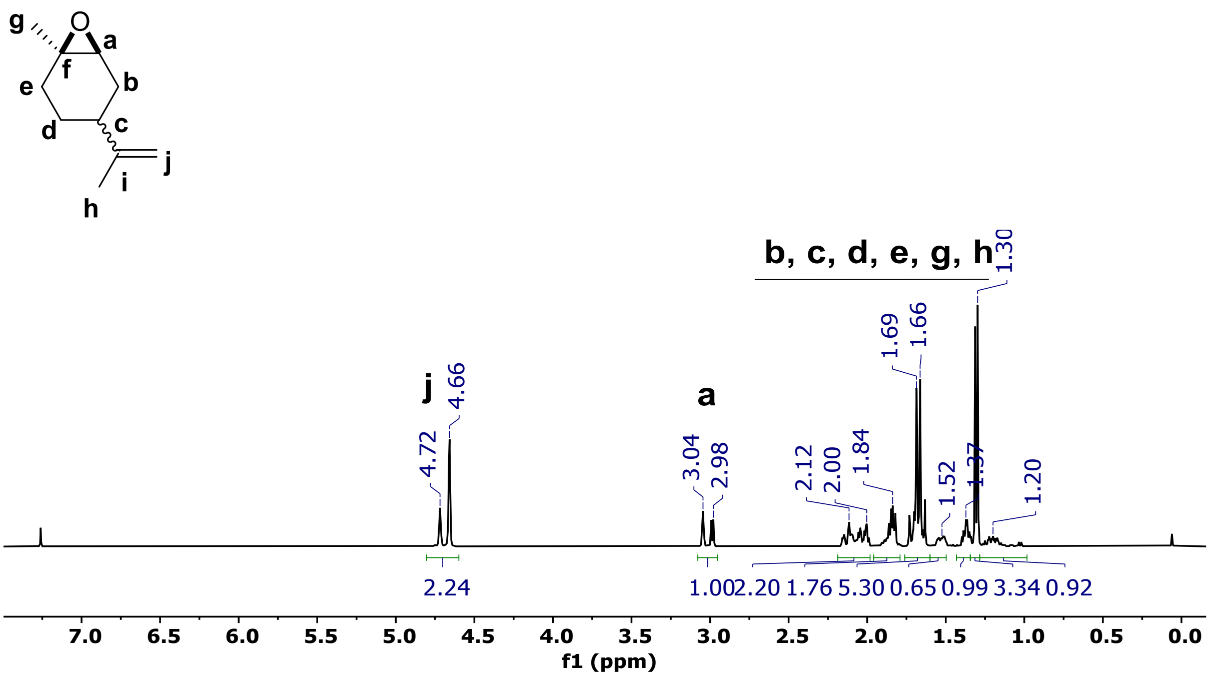

Fig. S29. ^{1}H NMR spectrum ($CDCl_3$) of LO isolated from the depolymerisation of PLC**.**

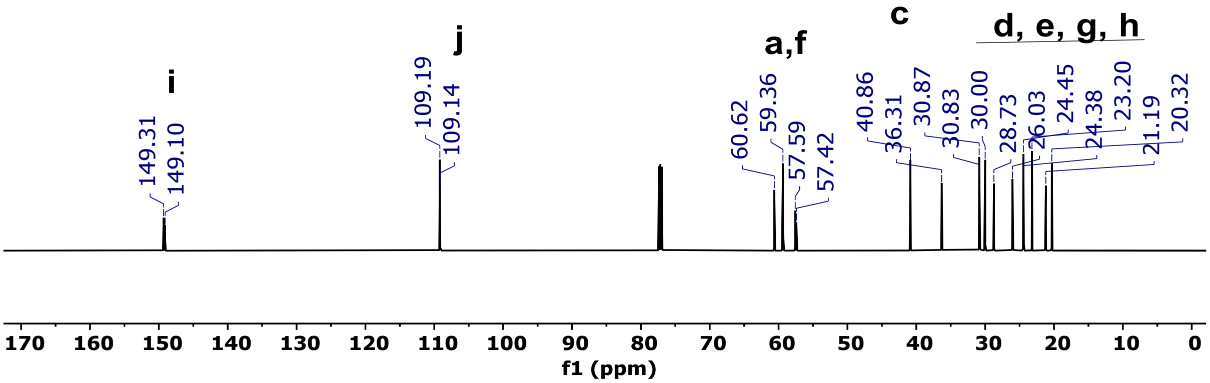

Fig. S30. ^{13}C {^{1}H} NMR spectrum ($CDCl_3$) of LO isolated from the depolymerisation of PCPC.

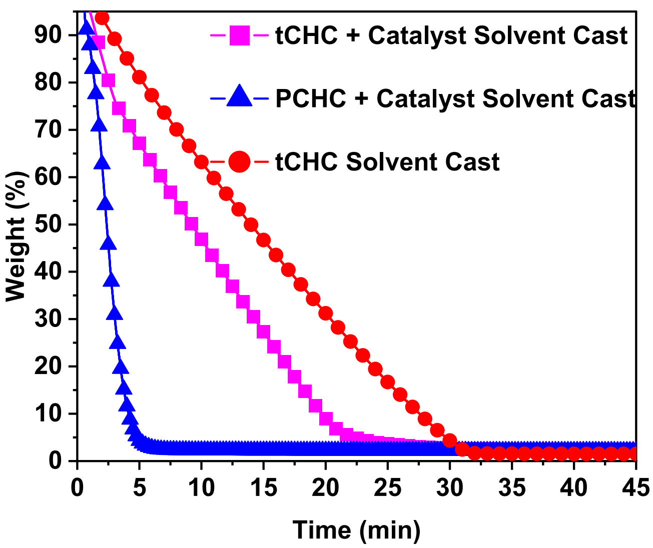

Fig. S31. TGA thermograms showing mass loss against time in mixtures of PCHC: Mg(II)Co(II) (300:1, blue triangles), *trans*-cyclohexene carbonate: Mg(II)Co(II) (300:1, purple squares) and *trans*-cyclohexene carbonate (red circles). The samples were prepared by solvent casting. The TGAs were run with a 60 minute isotherm at 140 °C.

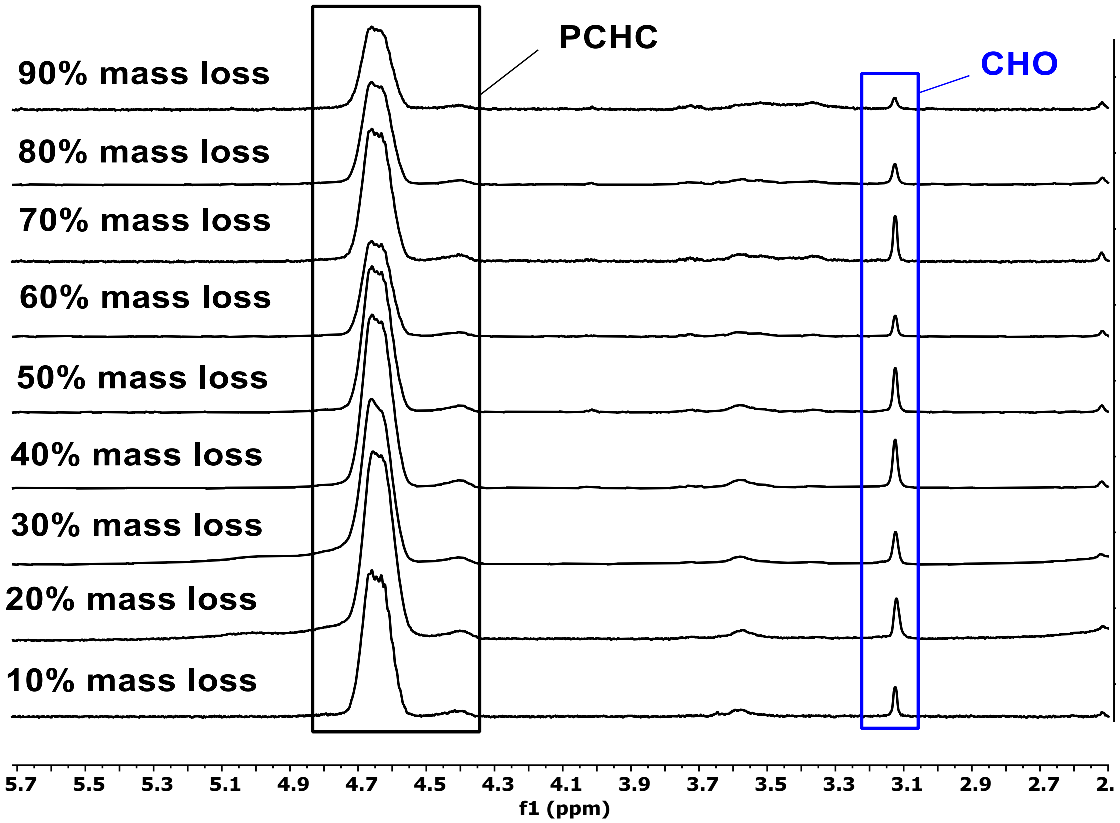

Fig. S32. ^{1}H NMR spectra ($CDCl_3$) of polymer:catalyst residue in TGA crucible for depolymerization at various %mass loss. (*trans*CHC expected at [illegible] = 3.98 ppm).

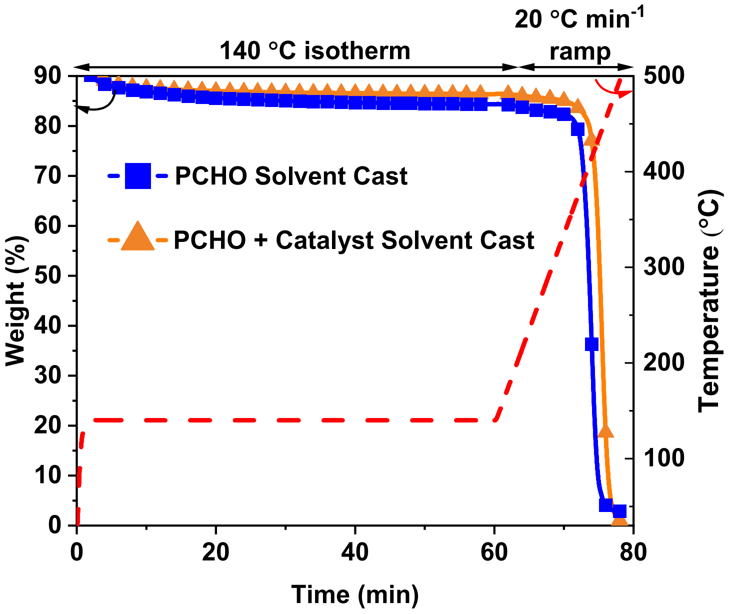

Fig. S33. TGA thermograms showing mass loss against time in a mixtures of PCHO: Mg(II)Co(II) (300:1, orange triangles) PCHO (blue squares) and the furnace temperature (red dashed line, right axis). The samples were prepared by solvent casting. The TGAs were run with an 60 minute isotherm at 140 °C followed by a 20 °C minute^{-1} ramp from 140 °C to 500 °C.

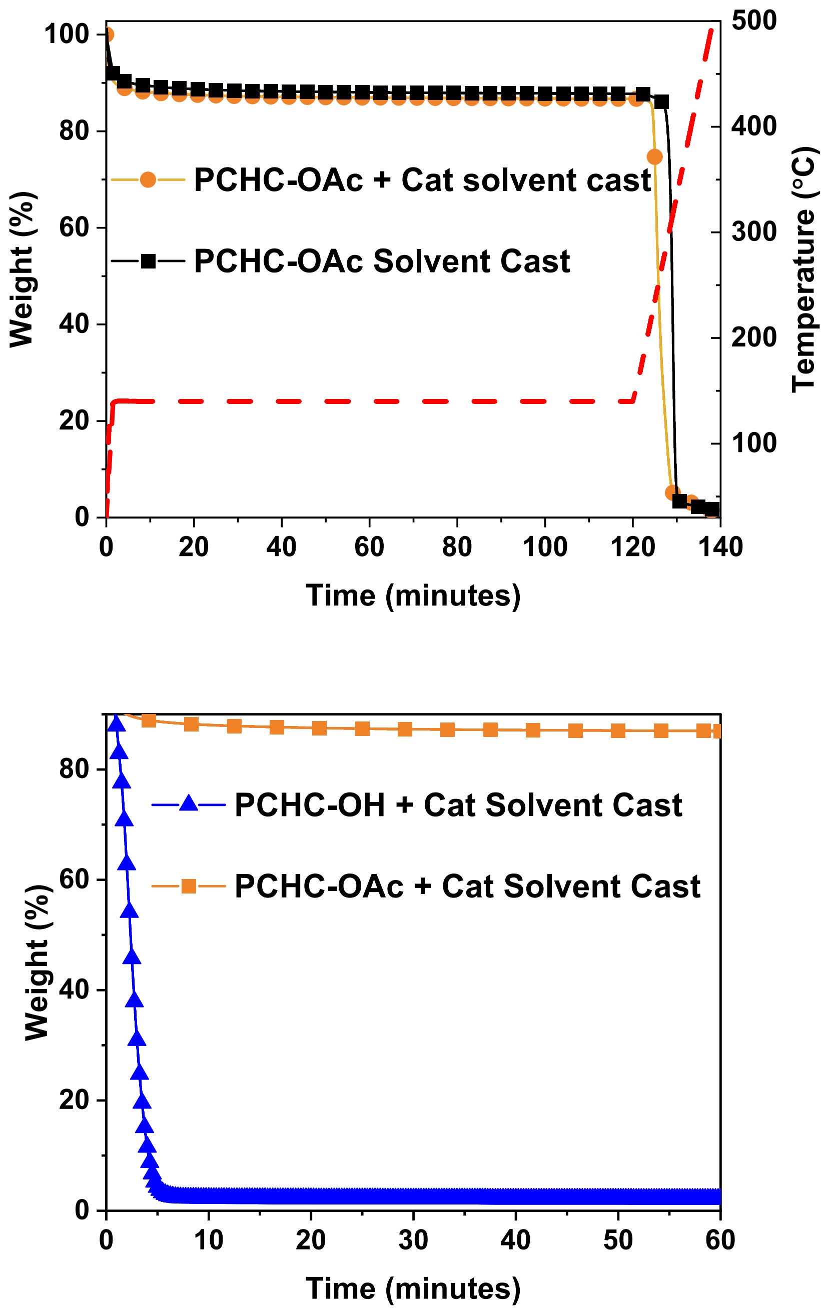

Fig. S34. The top thermogram shows mass loss against time in mixtures of acetyl end-capped PCHC (PCHC-OAc: Mg(II)Co(II) (300:1, orange circles) and PCHC-OAc (black squares) and the furnace temperature (red dashed line, right axis). The bottom themogram shows a comparison of hydroxyl-terminated and PCHC-OAc thermograms in the presence of Mg(II)(Co(II) (polymer:Mg(II)Co(II) = 300:1).The samples were prepared by solvent casting. The TGAs were run with an 120 minute isotherm at 140 °C followed by a 20 °C minute^{-1} ramp from 140 °C to 500 °C.

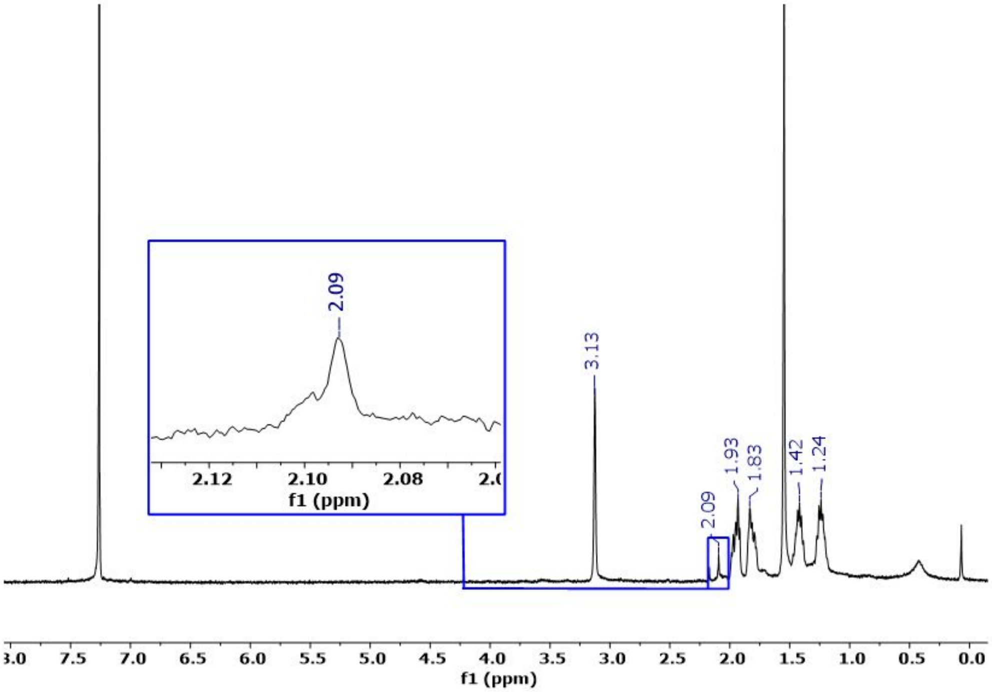

Fig. S35. ^{1}H NMR spectrum ($CDCl_3$) of CHO formed through Mg(II)Co(II) catalyzed depolymerization of PCHC (1:30, 140 °C). Acetic acid is detected at δ = 2.09 ppm (residual H_2O and $CHCl_3$ detected at δ = 1.51 ppm and 7.26 ppm, respectively).

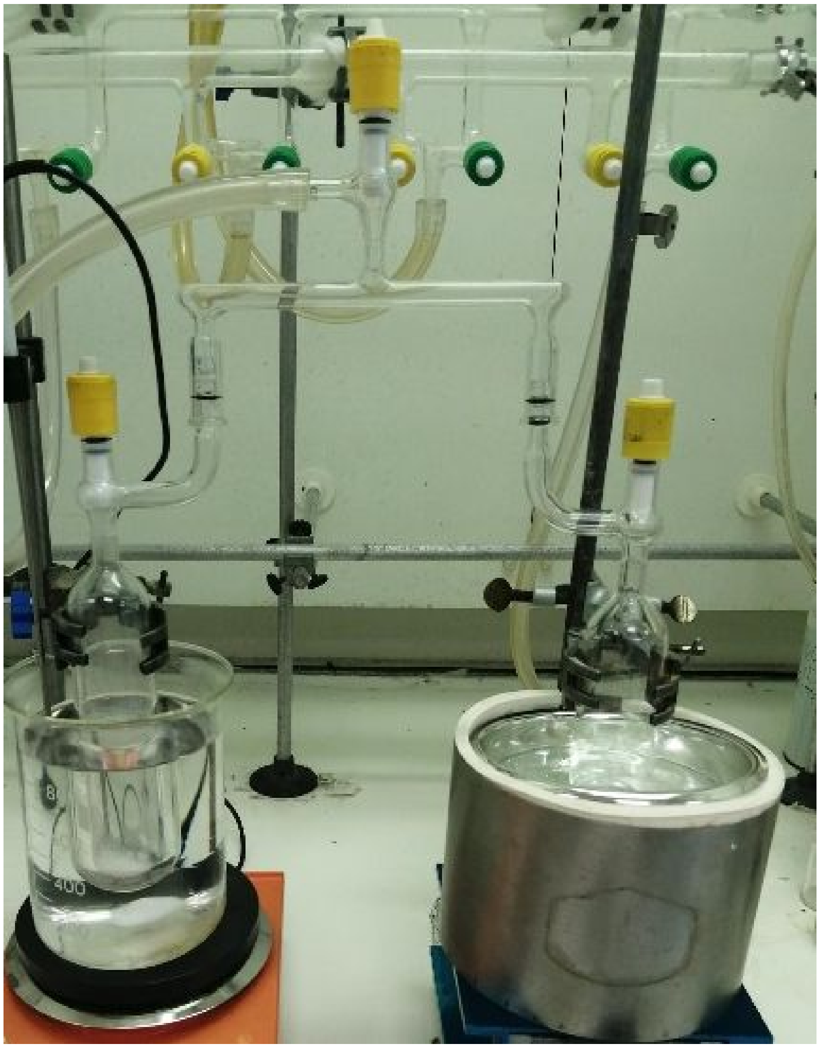

Fig. S36. Vacuum transfer apparatus. From left to right: flask continaing polymer and catalyst, T-junction and collection flask

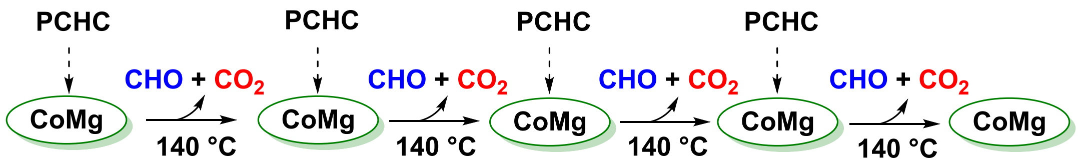

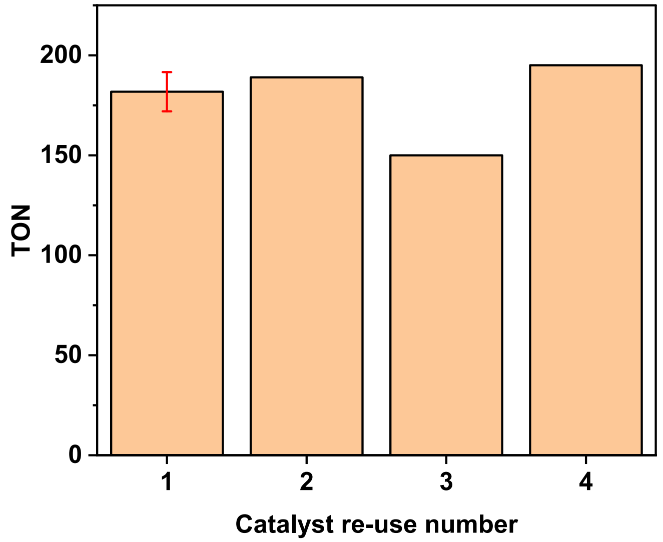

Fig. S37. Productivity of the catalyst over 4 reaction cycles.

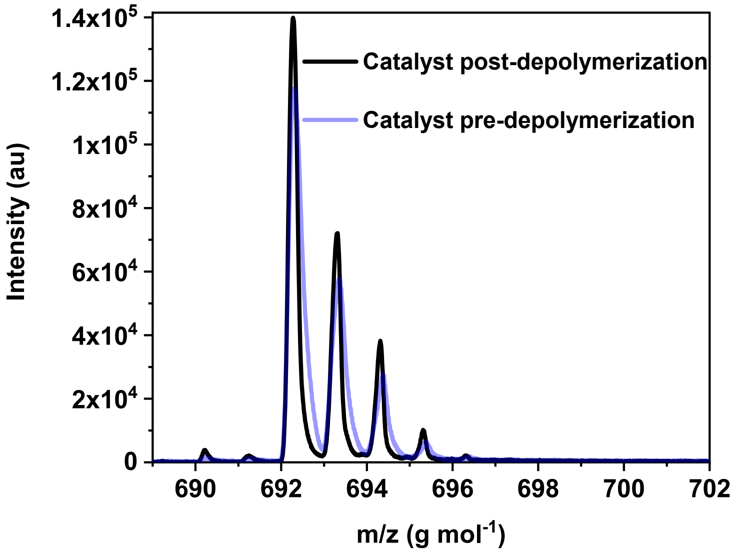

Fig. S38. MS (MALDI-TOF) spectrum of catalyst before (purple) and after (black) depolymerization. Ions were detected as [Mg(II)Co(II)L(OAc)]$^+$ species in positive reflector mode.

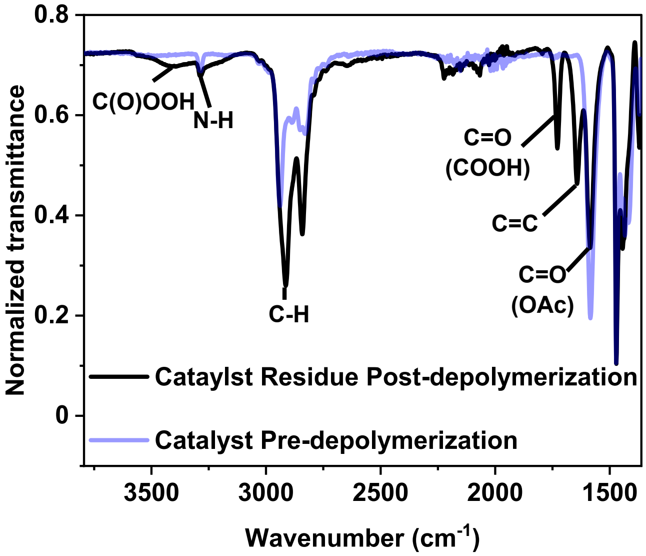

Fig. S39. IR spectra of Mg(II)Co(II) before (purple) and after (black) depolymerization. The spectra show that the NH (3290 cm^{-1}), CH (2841 cm^{-1}), and C=O (OAc, 1531cm^{-1}) stretches are unchanged post depolymerization, albeit with the complex exhibiting a less intense acetate stretch. Post depolymerization, new stretches appear at 3400 cm^{-1} (broad) 1728 cm^{-1} and 1640 cm^{-1}. These stretches do not correspond to residual polymer (1750 cm^{-1}), *trans*-CHC (1820 cm^{-1}) or *cis* CHC (1804 cm^{-1}) The stretches at 3400 cm^{-1} and 1728 cm^{-1} are thus attributed to a carboxylic acid species whilst the stretch at 1640 cm^{-1} remains unassigned..

Table S3. Depolymerisation of polycarbonates to isolate epoxides

Entry	Polymer	Time (h)	Bp epoxide (°C)	Temp. (°C)	Vac (mbar)	Yield (%)	Residual mass (%)
1[	PCHC	2	129	140	Static	92%	9%
2	PVCHC	2.5	169	140	200-25	79%	8%
3	PCPC	1	102	140	Static	95%	5%
4	PLC	5	203	140	200-50	74%	9%

Reactions were performed neat with $[\text{polymer}]_0$:$[\text{Mg(II)Co(II)}]_0$ 300:1 using 2.00 g of polymer using a distillation or or vacuum transfer apparatus.

Table S4. Polymerisation of epoxides formed through chemical recycling

Entry	Epoxide	Conv%[a]	%Polymer[a]	$M_{n,SEC}$ (g mol^{-1})[d]
1	CHO	62%	>99%	6700 (1.10)
2	VCHO	54%	>99%	10900 (1.08)
3[e]	CPO	38%	>99%	5000 (1.73)
4[f]	LO	54%	>99%	5700 (1.47)

Reactions performed in toluene at 100 °C with $[\text{epoxide}]_0$:$[\text{1,2-transcyclohexanediol}]_0$:$[\mathbf{2}]_0$ = 2000:10:1 and $[\text{epoxide}]_0$ = 2.9 M unless otherwise stated.[a] Determined by ^{1}H NMR spectroscopy using the relative integration of polycarbonate, cyclic carbonate and polyether against the epoxide.[c] Determined by ^{1}H NMR spectroscopy using the relative the polycarbonate, cyclic carbonate and polyether.[d] Calculated by SEC relative to polystyrene standards in tetrahydrofuran (THF) eluent; $Đ_M$=M_w/M_n.[e] $[\text{CPO}]_0$ = 4.3 M.[f] Reaction performed at 45 °C with $[\text{LO}]_0$:$[\text{Al(III)Tris}]_0$:$[\text{PPNCl}]_0$ = 100:2:1 with $[\text{LO}]_0$ = 3.8 M.

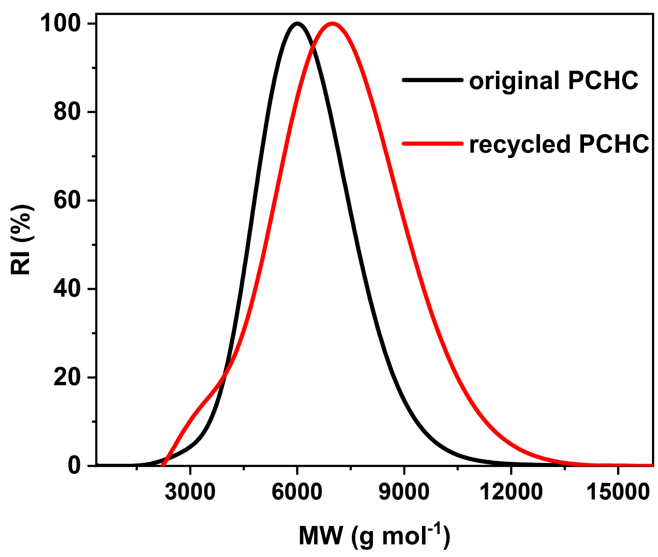

Fig. S40. SEC trace of original (black) and recycled (red) PCHC.

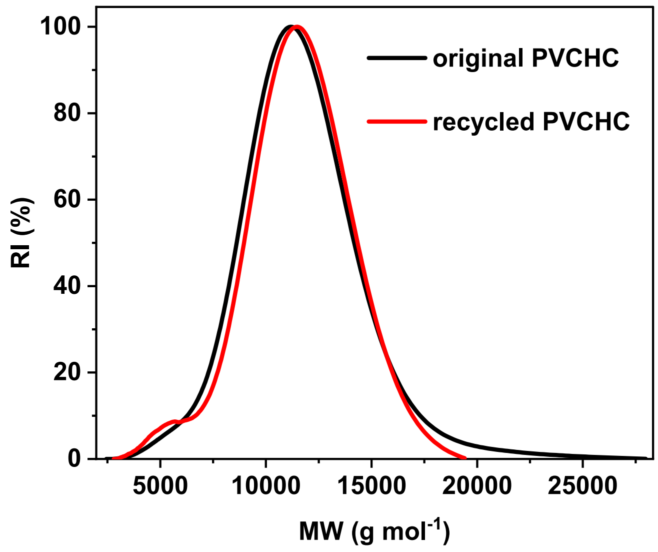

Fig. S41. SEC trace of original (black) and recycled (red) PVCHC.

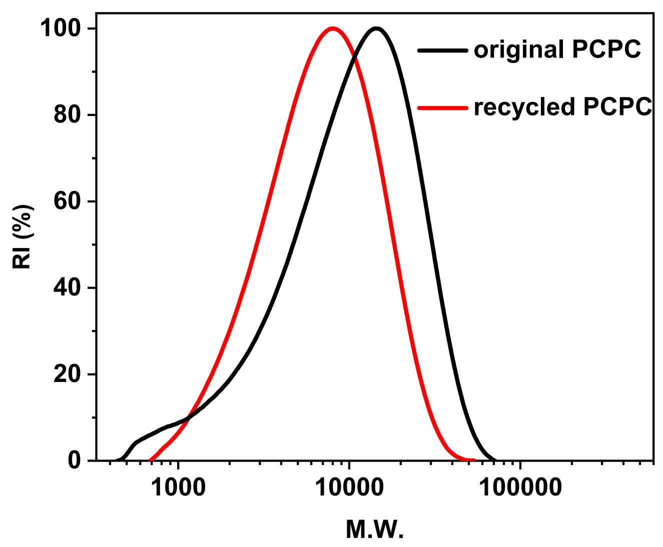

Fig. S42. SEC trace of original (black) and recycled (red) PCPC.

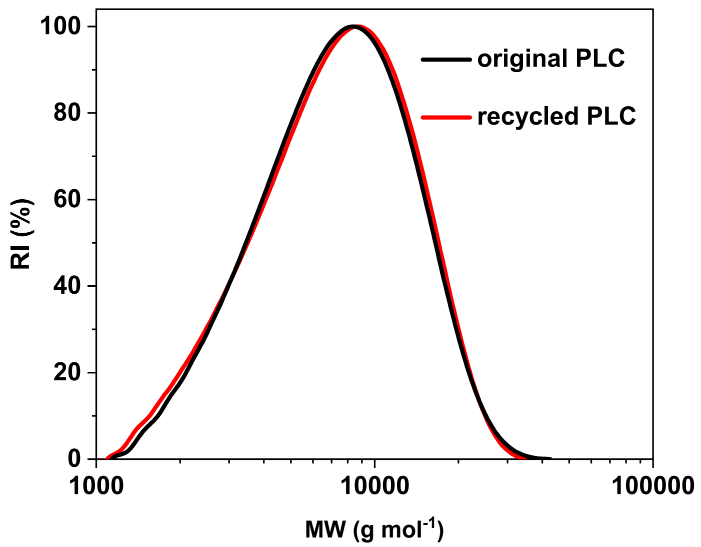

Fig. S43. SEC trace of original (black) and recycled (red) PLC.

5. NMR Characterisation of Polycarbonates

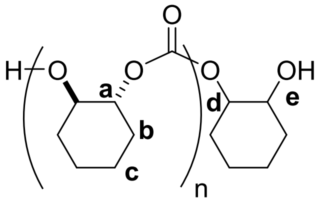

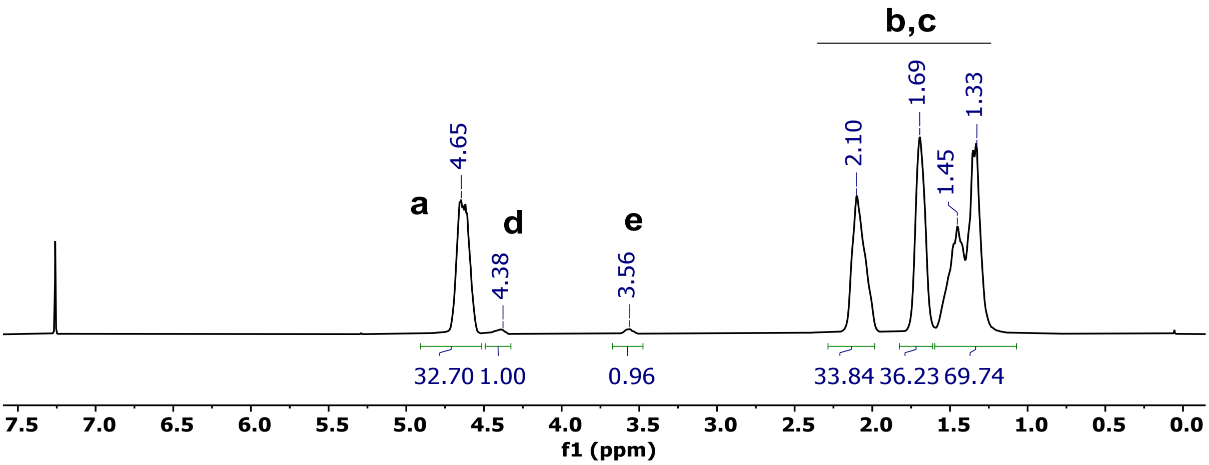

Fig. S44. ^{1}H NMR spectrum ($CDCl_3$) of PCHC.

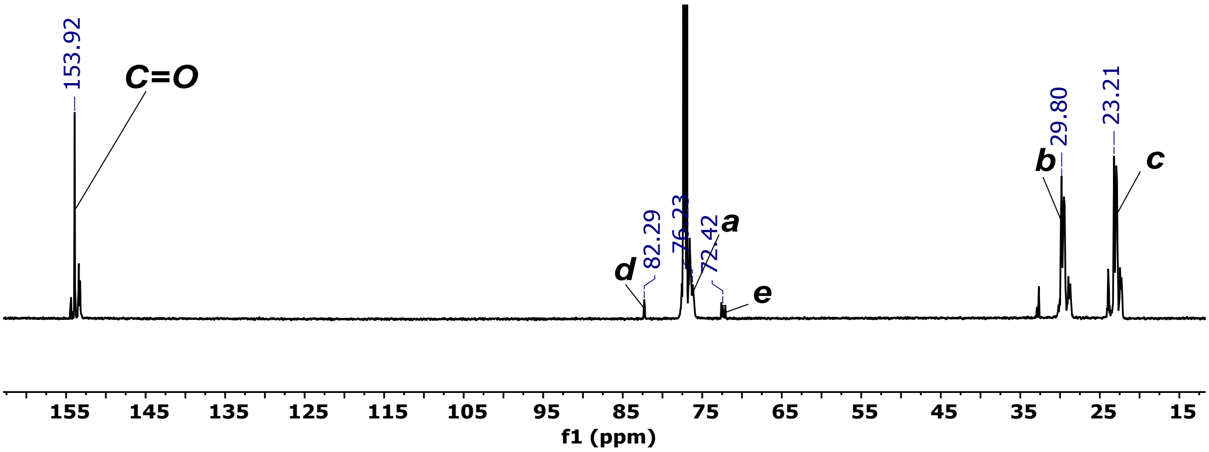

Fig. S45. ^{13}C {^{1}H} NMR spectrum ($CDCl_3$) of PCHC.

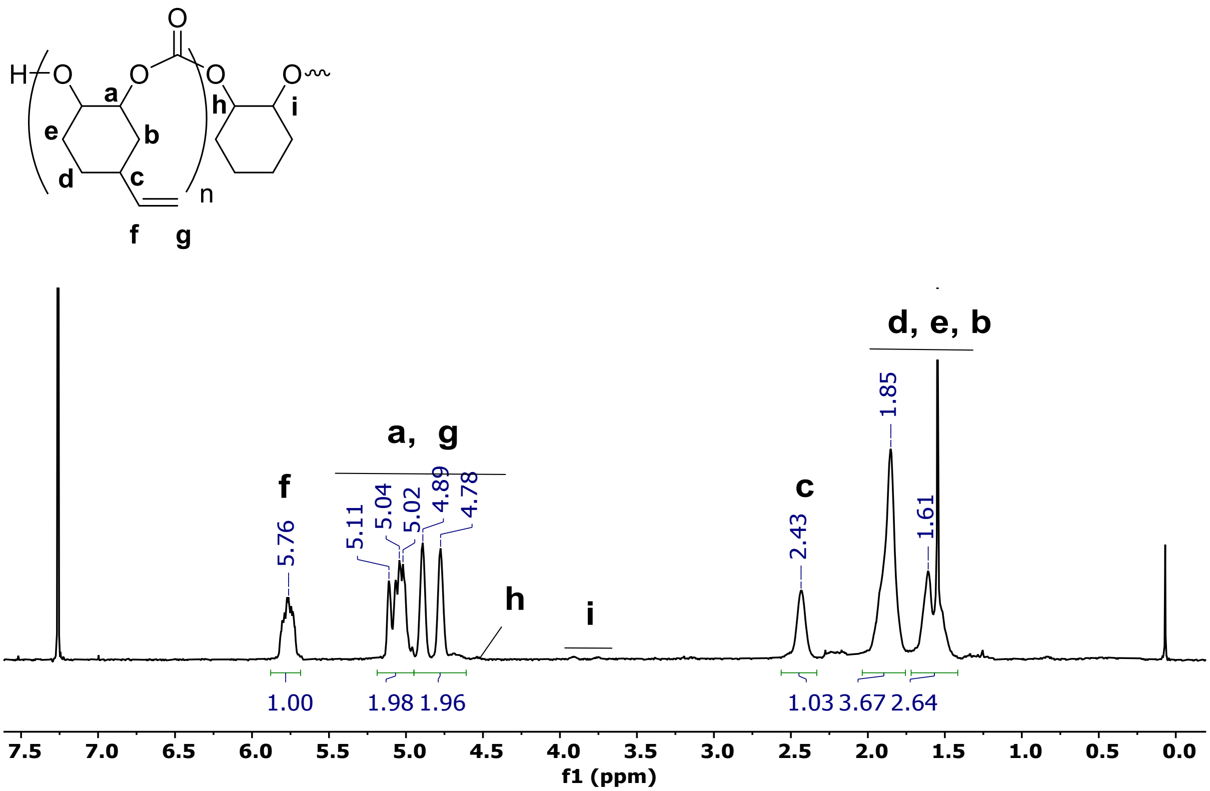

Fig. S46. ^{1}H NMR spectrum ($CDCl_3$) of PVCHC.

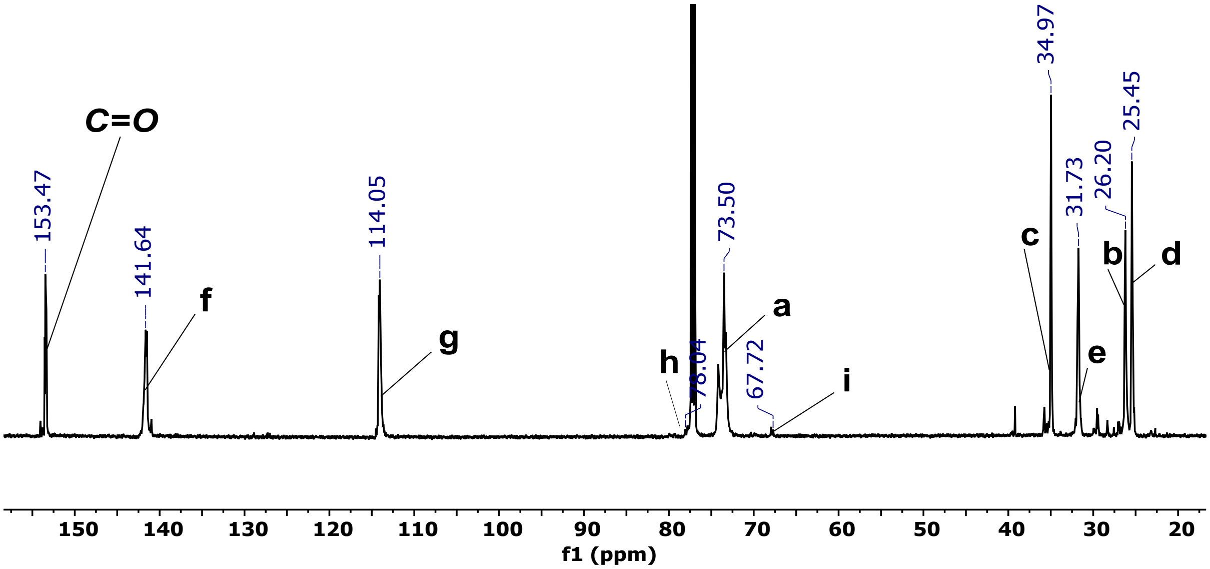

Fig. S47. ^{13}C $\{^{1}H\}$ NMR spectrum ($CDCl_3$) of PVCHC.

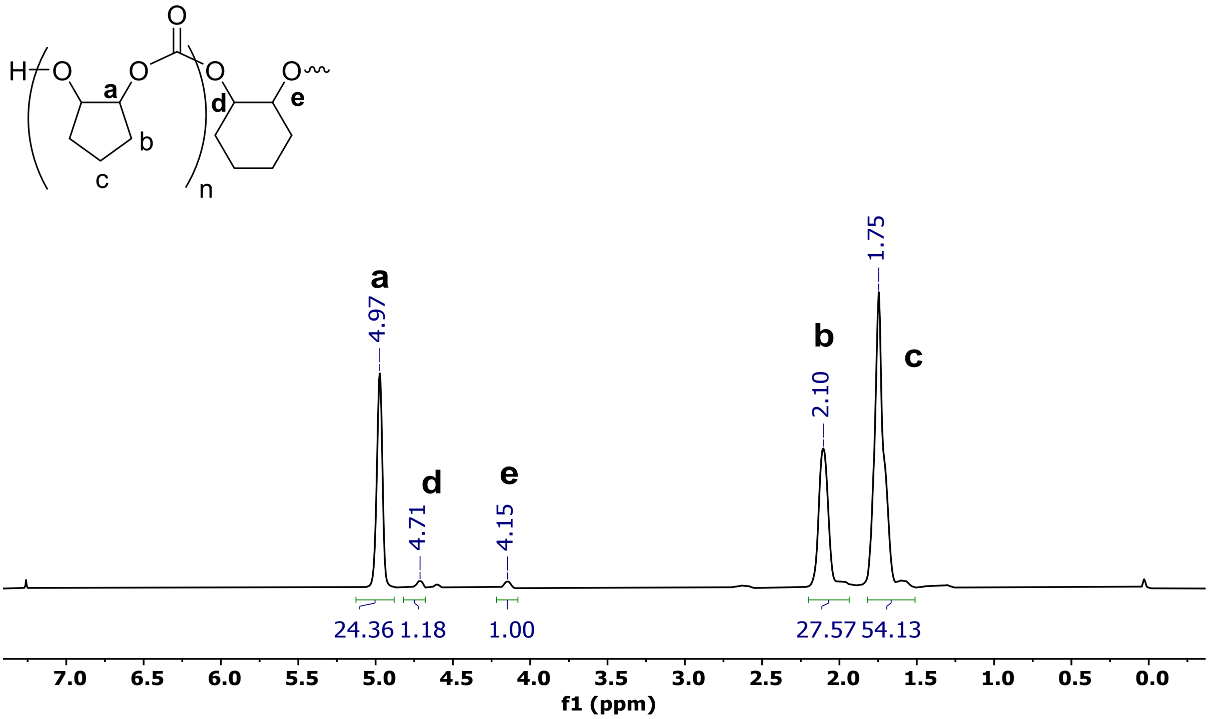

Fig. S48. ^{1}H NMR spectrum ($CDCl_3$) of PCPC.

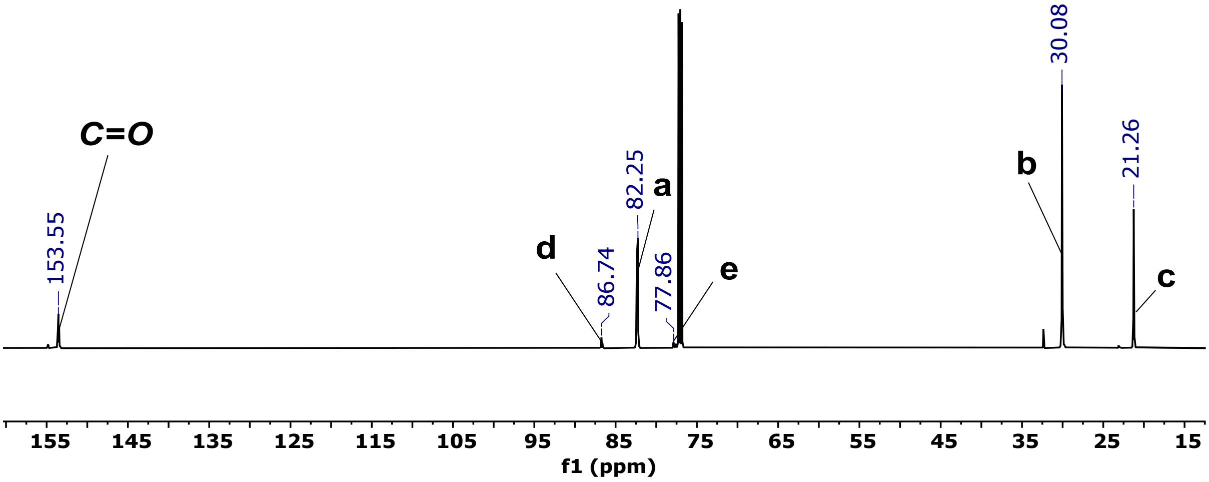

Fig. S49. ^{13}C {^{1}H} NMR spectrum ($CDCl_3$) of PCPC.

.

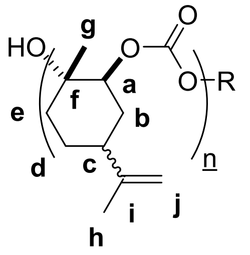

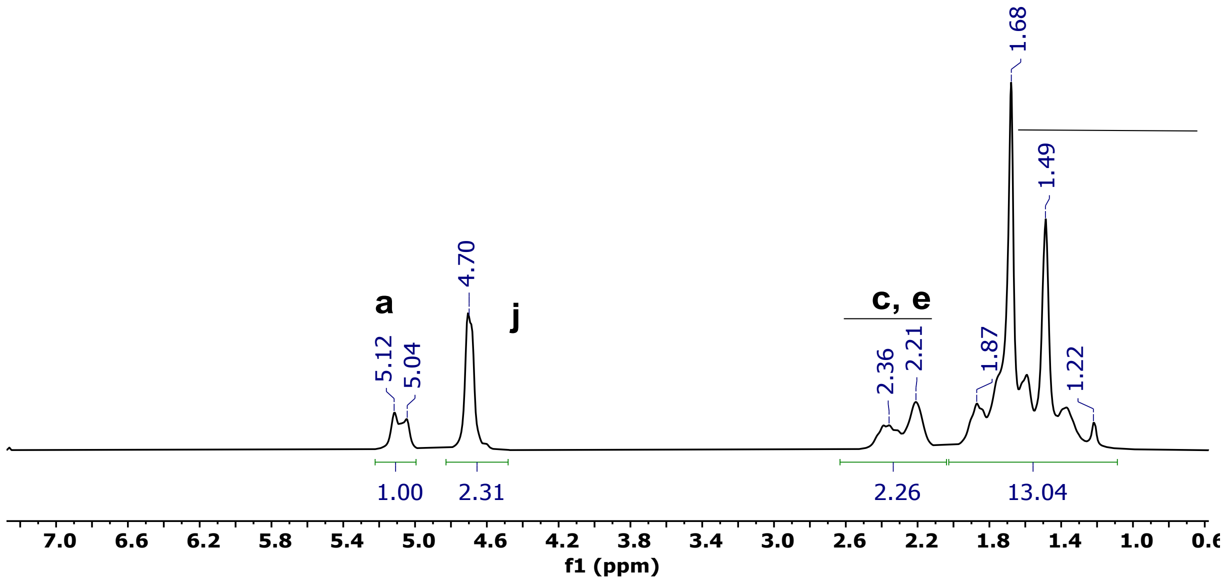

Fig. S50. ^{1}H NMR spectrum ($CDCl_3$) of PLC.

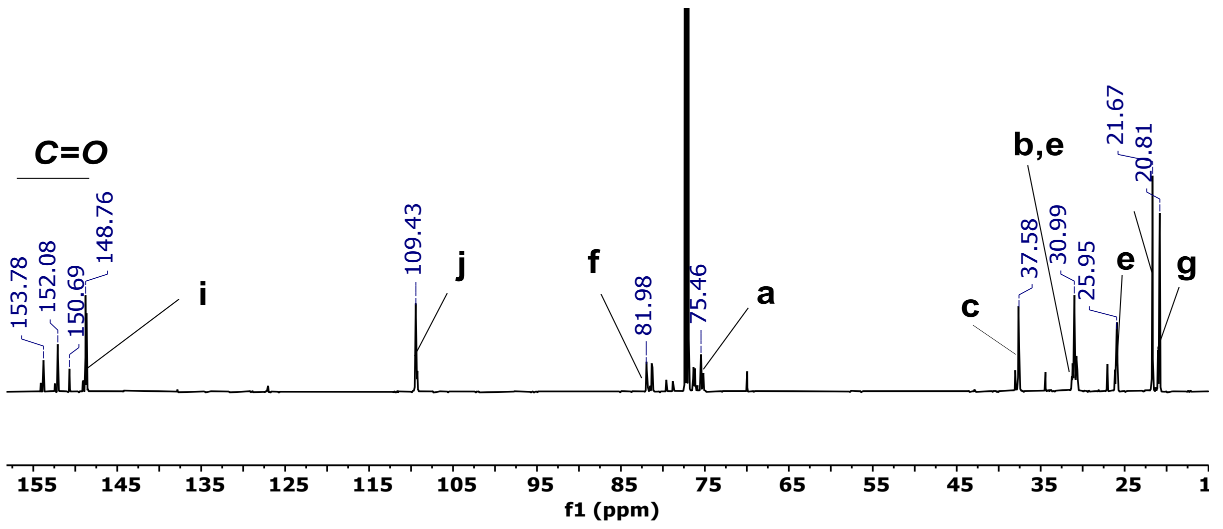

Fig. S51. ^{13}C {^{1}H} NMR spectrum ($CDCl_3$) of PLC.

8. References

[1] M. R. Kember, P. D. Knight, P. T. R. Reung, C. K. Williams, *Angew. Chem. Int. Ed.* **2009**, *48*, 931-933.

[2] M. R. Kember, C. K. Williams, *J. Am. Chem. Soc.* **2012**, *134*, 15676-15679.

[3] A. C. Deacy, A. F. R. Kilpatrick, A. Regoutz, C. K. Williams, *Nat. Chem.* **2020**, *12*, 372-380.

[4] A. C. Deacy, E. Moreby, A. Phanopoulos, C. K. Williams, *J. Am. Chem. Soc.* **2020**, *142*, 19150-19160.

[5] C. J. Whiteoak, N. Kielland, V. Laserna, E. C. Escudero-Adán, E. Martin, A. W. Kleij, *J. Am. Chem. Soc.* **2013**, *135*, 1228-1231.
